# Factors influencing implementation of interventions to promote birth preparedness and complication readiness

**DOI:** 10.1186/s12884-017-1448-8

**Published:** 2017-08-31

**Authors:** Andrea Solnes Miltenburg, Yadira Roggeveen, Jos van Roosmalen, Helen Smith

**Affiliations:** 1 Department of Community Medicine, Institute of Health and Society, University of Oslo, P.O. Box 1072, Blindern, 0316 Oslo, Norway; 2 Athena Institute for Research on Innovation and Communication in Health and Life sciences, Faculty of Earth and Life Sciences, VU University, De Boelelaan 1105, 1081 HV Amsterdam, Netherlands; 3 Centre for Maternal and Newborn Health, Liverpool School of Tropical Medicine, Pembroke Place, Liverpool, L3 5QA UK

**Keywords:** Birth preparedness and complication readiness, Maternal health, Skilled birth attendant

## Abstract

**Background:**

The recent WHO report on health promotion interventions for maternal and newborn health recommends birth preparedness and complications readiness interventions to increase the use of skilled care at birth and to increase timely use of facility care for obstetric and newborn complications. However, these interventions are complex and relate strongly to the context in which they are implemented. In this article we explore factors to consider when implementing these interventions.

**Methods:**

This paper reports a secondary analysis of 64 studies on birth preparedness and complication readiness interventions identified through a systematic review and updated searches. Analysis was performed using the Supporting the Use of Research Evidence (SURE) framework to guide thematic analysis of barriers and facilitators for implementation.

**Results:**

Differences in definitions, indicators and evaluation strategies of birth preparedness and complication readiness interventions complicate the analysis. Although most studies focus on women as the main target group, multi-stakeholder participation with interventions occurring simultaneously at both community and facility level facilitated the impact on seeking skilled care at birth. Increase in formal education for women most likely contributed positively to results. Women and their families adhering to traditional beliefs, (human) resource scarcities, financial constraints of women and families and mismatches between offered and desired maternity care services were identified as key barriers for implementation.

**Conclusions:**

Implementation of birth preparedness and complication readiness to improve the use of skilled care at birth can be facilitated by contextualizing interventions through multi-stakeholder involvement, targeting interventions at multiple levels of the health system and ensuring interventions and program messages are consistent with local knowledge and practices and the capabilities of the health system.

**Electronic supplementary material:**

The online version of this article (doi:10.1186/s12884-017-1448-8) contains supplementary material, which is available to authorized users.

## Background

Our systematic review on the impact of birth preparedness and complication readiness (BPCR) interventions on birth with a skilled attendant revealed that BPCR is a complex intervention, highly dependent on the context in which it is implemented [1]. We also found that BPCR interventions vary in terms of approaches, actors involved, in definitions applied, in outcomes measured and in the strategies used to evaluate them.

The concept of BPCR emerged almost 20 years ago and is described as a process of planning for birth and anticipating actions in case of obstetric emergencies in order to reduce delays in seeking skilled care [2]. In 2005 BPCR was included in the World Health Organization (WHO) antenatal care package [3, 4], with emphasis on the following elements: deciding on desired place of birth; preferred birth attendant; location of the closest facility for birth and in case of complications: funds for expenses related to birth and/or complications; supplies necessary to bring to the facility; an identified labour and birth companion; an identified support to look after home and other children while the woman is away; transport to a facility for birth or when complications arise; and identification of compatible blood donors when needed. At around the same time, Johns Hopkins Program for International Education in Gynecology and Obstetrics (JHPIEGO) developed a BPCR matrix acknowledging the important role of coordinated efforts of all ‘safe motherhood stakeholders’ for implementing BPCR. The matrix delineates roles and activities of policymakers, facility managers, providers, communities, families, and women in ensuring that women and newborns reach accessible, appropriate, acceptable and good quality care during pregnancy, childbirth and postpartum [2].

Despite widespread promotion and inclusion of BPCR in Safe Motherhood interventions, evidence on the effect of BPCR interventions remains limited. Our recent systematic review of the available evidence found that BPCR, as part of a package of interventions, has the potential to increase skilled care at birth and timely use of facility care for obstetric and newborn complications [1]. The results of the review have been included in recently published WHO guidelines on health promotion interventions for maternal and newborn health, where WHO recommends implementation of BPCR interventions [5].

To support those who plan to implement BPCR interventions, we conducted a secondary analysis of the papers included in our systematic review [1] and additional studies identified, in order to identify factors influencing implementation. We explore stakeholder perceptions and experiences of BPCR interventions, identify barriers and facilitators to BPCR implementation, and discuss how these relate to improvements in use of skilled care at birth.

## Methods

This article reports a secondary analysis of studies identified in a systematic review conducted in 2013 [1, 6] and additional articles identified through a subsequent search. The systematic review included articles published in English between 2000–2012, identified from PubMed, Embase and CINAHL plus a manual search of the grey literature and a database that included results of systematic mapping of maternal health research in low- and middle-income countries [7]. The original review was concerned with effects on care seeking including use of a skilled attendant at birth (SBA) or facility birth, use of antenatal care (ANC) as well as effects on knowledge and preparations made for BPCR.

For this secondary analysis of factors influencing implementation, we included all articles included in the systematic review [*n* = 33] of 20 BPCR interventions. Additional identified studies include 16 papers on BPCR consisting mainly of descriptive studies [8–23] and a methodological evaluation of BPCR [24] which were identified through the original search but excluded for the systematic review. A subsequent search identified 14 newly published studies of the past 3 years for inclusion in this article [25–38]. In total we reviewed 64 papers for this secondary analysis.

For the findings presented in this paper, we conducted a narrative synthesis of qualitative information on implementation factors from the 64 papers. We used an adapted SURE (Supporting the Use of Research Evidence) framework to guide the extraction of relevant information from studies and to structure the synthesis [39]. The framework comprises a comprehensive list of barriers and facilitators to implementing health systems interventions including stakeholder knowledge and attitudes, health service delivery factors, and social and political considerations; the framework has been used in other systematic reviews of qualitative evidence [40, 41].

## Results

### Description of included studies

Characteristics of the 64 included studies are presented in Table 1; some studies report on the same BPCR programme or intervention and are listed together. Most of the studies of BPCR interventions were conducted in South Asia (Nepal *n* = 7; India *n* = 6; Bangladesh *n* = 4; Pakistan *n* = 1; and Tibet *n* = 1), followed by East Africa (Tanzania *n* = 7; Ethiopia *n* = 6; Uganda *n* = 4; Eritrea *n* = 1; and Kenya *n* = 1), West Africa (Burkina Faso *n* = 3; Nigeria *n* = 3; and Benin *n* = 1), South East Asia (Cambodia *n* = 1; and Indonesia *n* = 1), and Latin America (Guatemala *n* = 1); one study included multiple countries, and two articles were literature reviews. BPCR implementation strategies varied and often included multiple interventions, which are summarized in Table 2. These included house visits by volunteers who provided education on BPCR, training of health workers in facilities to provide BPCR as part of ANC, provision of education materials or other visual aids with BPCR information, community mobilization activities to increase awareness on BPCR and mass media campaigns with BPCR messages.

**Table 1:** Characteristics of included studies (^a^studies included in the systematic review)

Author(s)	Study Design	Setting	Description of intervention
Ahluwalia et al., 2003; Kaharuza, 2001; Ahluwalia et al., 2010; ^a^	Pre and post study with qualitative component	TANZANIA, rural, Kwimba and Missungwi districts, Mwanza region	Multiple interventions included house visits by volunteers to provide BPCR education to pregnant women and their families. A community surveillance system for pregnancies and community-based plans for transportation to health facilities were also set up. Transport methods included by canoe, oxcart, bicycle/tricycle and stretcher.
Acharya et al., 2015	Cross-sectional facility based study	INDIA, New Delhi	**Study aim**: To assess the status of BPCR among pregnant women attending a primary health center. **Main findings**: Majority of women had identified a skilled attendant at birth for delivery. Nearly half of the women had saved money for delivery and had also identified a mode of transportation for the delivery.
Agarwal 2010	Cross-sectional study	INDIA, Indore City (Slum)	**Study aim:** To present levels and factors associated with BPCR among slum women. **Main findings:** Half of all respondents were well prepared taking more than 2 steps (identified a trained birth attendant, identified a health facility, arranged for transport, and saved money for emergency). Factors associated with well prepared women were maternal literacy and availing of antenatal services. A TBA attended majority of births. Skilled attendance during delivery was three times higher in well-prepared mothers compared to less-prepared mothers.
August 2015	Cross-sectional study	TANZANIA, Rufiji District	**Study aim**: to assess men’s knowledge and their involvement in BPCR. **Main findings**: Half of the men were able to mention one danger sign and made birth preparations in the form of a birth kit. There was no association with age, education and marital status.
Baqui et al. 2008^a^	Quasi-experimental pre and post comparative study with a control group	INDIA, rural districts of Uttar Pradesh	Interventions included house visits by auxiliary nurse midwives (ANMs) or Anganwadi workers and change agents to provide BPCR counselling to pregnant women and their families.
Brazier et al., 2009; Hounton et al., 2008; Graham et al., 2008); Hounton et al., 2008; Newlands, 2008; Graham et al., 2008^a^	Quasi-experimental pre and post study with a control group	BURKINA FASO, rural	Behaviour change and community mobilization through participatory theatre and songs. Upgrading of health facilities and improving the referral system. Control district was also provided with facility upgrades but not the behavioural change component.
Choudhury 2011	Qualitative	BANGLADESH, Rangpur and Kurigram districts	**Study aim:** qualitative exploration of the existing maternal care practices during pregnancy, delivery and post-partum period among women of the ultra poor households. **Main findings:** Traditional believes could impose restrictions in mobility (evil spirits more active in evening). Few women were birth prepared and local birth attendant (TBA) was not contacted in advance out of fear of black magic. Making too many plans for arrival such as new clothes could bring bad luck. There was fear of health workers for various reasons.
Darmstadt et al., 2010^a^	Cluster-RCT	BANGLADESH, rural unions in Mirzapur	Antenatal house visits by volunteers who promoted BPCR including for newborn education. CHWs conducted additional postnatal visits to promote preventive newborn care practices and to identify and refer sick neonates. The control group received the usual care services provided by the local and national governments.
Debelew 2014	Cross-sectional study	ETHIOPIA, Jimma Zone	**Study aim:** to identify the factors affecting birth preparedness and complication readiness at the different levels. **Main findings:** The majority of respondents planned to save money and to arrange transport. One third planned to give birth in health facility and planned to be attended by skilled attendant for their current pregnancy. Being in urban residence and having health center within two hours distance as well as educational status of primary or above, husband’s occupation of employed or merchant, third or above wealth quintiles, knowledge of key danger signs during labor and attitude and frequency of antenatal care visits increased the likelihood of preparation for birth and its complications.
Dickerson 2010	Programme evaluation	TIBET, Medrogongkar and Dulung Dechen County	**Intervention:** PAVOT (Pregnancy and Village Outreach Tibet) is a community- and home-based maternal- newborn outreach program that serves rural pregnant Tibetan women. The program is based on a training-of- trainers model in which experienced master trainers train rural healthcare workers and laypersons to outreach the homes of rural-living women and families. During out- reach, providers relay maternal-newborn health education, hands-on skills training, and material resources directly to recipients. One of the specific PAVOT interventions was encouragement of development of a birth plan. **Main findings:** The majority of women attended more than 3 ANC visits but half of the women gave birth at home.
Ekabua 2011	Cross-sectional study	NIGERIA, Cross River State	**Study aim:** to assess the awareness and intention to use maternity services **Main findings**: The majority of respondents were aware of birth preparedness. Knowledge of danger signs was poor.
Family Care International Kenya, 2007; Moor et al., 2002(; Family Care International Kenya, 2003^a^	Quasi-experimental pre and post study	KENYA, Homa Bay and Migori districts, Nyanza province	Behaviour change campaign making use of printed materials including birth preparedness messages through drama and meetings. Materials supplied to health care workers (HCWs) as well as facility upgrades and improving provider skills. The control district received only facility intervention.
Family Care International Tanzania, 2007^a^	Quasi-experimental pre and post study with a control group	TANZANIA, Igunga and Urambo districts, Tabora region	Behaviour change communication and mobilization efforts through participatory meetings at village level and theatre and performing arts. Improvements to the availability and quality of maternity care through strengthening physical infrastructure and improving provider skills. Control district received no intervention.
Fonseca-Becker et al., 2004^a^	One group before and after evaluation	GUATEMALA, north- west and south-west regions	Service delivery improvements and trained health care providers and behaviour change interventions focused on organizing communities to effectively respond to obstetric emergencies and creating demand for the improved services through the use of radio and printed materials. Includes a community action cycle: a five-step participatory cycle consisting of organizing for community action; promoting community dialogue; planning together; collective action; and participatory evaluation. The communities developed their own emergency plans.
Hailu 2011	Cross-sectional study	ETHIOPIA, Sidama Zone	**Study aim:** To assess the current status and factors associated with birth preparedness and complication readiness among pregnant women. **Main findings:** The majority prepared to give birth at home and few women were birth prepared. First time pregnancy and ANC attendance were associated with being prepared.
Hiluf 2007	Cross-sectional study	ETHIOPIA, Tigray Region	**Study aim**: to assess knowledge and practices with respect to BPCR and factors associated with women who gave birth in the last 12 months. **Main findings**: Nearly a quarter of the respondents were prepared for birth and its complications. Preparedness was higher amongst literate mother, married women, women with previous stillbirth and those who received information about BPCR.
Hodgins et al., 2009; Valley Research Group 2007^a^	One group before and after evaluation	NEPAL, rural, Jhapa and Banke districts	House visits by volunteers who provide BPCR education to pregnant women and family members, making use of pictorial handouts.
Hossain et al., 2006; Barbey et al., 2001^a^	Quasi-experimental study pre and post comparative study with a control group	BANGLADESH, rural, Birampur region	Interventions included facility upgrades, quality of care and BPCR and community mobilization. SBAs, fieldworkers and village doctors were trained to disseminate BPCR messages that were also incorporated into a variety of visual aids during home visits, group discussions at clinics and village meetings. Comparison district received facility upgrade but no community intervention; control district received no intervention.
Iliyasu 2010	Cross-sectional study	NIGERIA, Kano State	**Study aim**: to assess men’s perception of high risk pregnancy and danger signs; birth preparedness and complication readiness, and participation in maternity care. **Main findings**: Half of the men considered bleeding a danger sign. One third mentioned convulsion as danger sign. Les than a third of the men made arrangements for mother’s health care, transportation and delivery or made savings for obstetric emergencies. One third of the men accompanied women to maternity care. Higher participation was observed in younger educated men.
Jennings 2010	Quasi-experimental pre and post comparative study with a control group	BENIN, Zou/Collines region	**Intervention**: Introduction of the job aids: a set of pictorial counseling cards designed to support communication to women about care during and after pregnancy according to national guidelines. Intervention components: training, organizational changes, and field support. All health care personnel at the intervention sites were trained for three days in the content and use of the counseling cards, interpersonal communication, and quality improvement. **Main findings:** The study measured three outcomes: (1) quality of counseling provided to pregnant women; (2) provider perceptions regarding use of the job aids; and (3) women’s knowledge of messages relating to maternal and newborn care. Women in the intervention arm received more recommended messages than in the control arm. Increased communication skills regarding use of visual aids and verification of understanding was seen in the intervention arm. Improvements in knowledge among pregnant women were observed in the area of birth preparedness, recognition of danger signs, and clean delivery.
Kabakyenga 2011	Cross-sectional survey	UGANDA, Mbarara district	**Study aim:** To explore the association between knowledge of obstetric danger signs and birth preparedness among recently delivered women: **Main findings**: More than half of the women knew at least one danger signs during pregnancy, childbirth than during the post-partum period. Few women had knowledge of 3 or more key danger signs during the three periods. Of the four birth preparedness practices; 91% had saved money, 71% had bought birth materials, 61% identified a health professional and 61% identified means of transport. Overall one third of the respondents were birth prepared (saved money, bought materials, identified health professional and identified transport). Young age and high levels of education had synergistic effect on the relationship between knowledge and birth preparedness.
Kabakyenga 2012	Cross-sectional survey	UGANDA, Mbarara district	**Study aim:** to assess the influence of birth preparedness practices and decision-making on location of birth and assistance by SBAs. **Main findings**: One third of the women had been prepared for childbirth and the prevalence of assistance by SBAs in the sample was two thirds. Decision making on location of birth was the husband in the majority of cases. When women made the final decision on location of birth in consultation with either the spouse or other people, the likelihood of giving birth assisted by a skilled birth attendant was very high and low when they made the decision alone.
Kakaire 2011	Cross-sectional facility based study	UGANDA, Kabale district	**Study aim:** to assess factors associated with birth preparedness and complication readiness as well as the level of male participation in the birth plan and healthcare seeking for emergency obstetric referrals. **Main findings**: Nearly half of the women had saved money in the event of complications and were joined by their men to ANC and during labour.
Karkee 2013	Prospective cohort study	NEPAL, Kaski district	**Study aim:** to assess birth preparedness level in expectant mothers and to evaluate its association with skilled attendance at birth. **Main findings**: The majority of women were birth prepared, 72% prepared the five activities (identification of delivery place, identification of transport, identification of blood donor, money saving, and antenatal care check-up). Of the cohort 85% SBA and it appeared that the more arrangements made, the more likely were the women to have skilled attendance at birth.
Kaso 2014	Cross-sectional study	ETHIOPIA, Robe Woreda, Oromia Region	**Study aim:** to assess knowledge and practices with respect to birth preparedness and complication readiness and factors associated in rural community among women of reproductive age. **Main findings**: Few respondents were prepared for birth and its complications and was higher amongst educated women.
Kumar et al., 2012; Kumar et al., 2008^a^	Cluster-RCT	INDIA, rural Shivgarh, Uttar Pradesh	Intervention package including home visits, community meetings and folk-song meetings, maternal and newborn health stakeholder meetings and meetings for community volunteers. Control clusters received standard care.
Kuteyi 2011	Cross-sectional study	NIGERIA, Osun State	**Study Aim:** to assess knowledge and practices of pregnant women attending antenatal clinics with respect to BPCR. **Main findings:** The majority of pregnant women had poor knowledge of obstetric danger signs; only a third were birth prepared (if they had identified and agreed on a place of delivery, were saving money towards delivery, had begun purchasing materials/supplies for a clean delivery and newborn care, if they knew their estimated date of delivery and had undergone voluntary counseling and testing for HIV), while one third were not complication ready (if they fulfilled at least four of the following criteria: had adequate knowledge of danger signs as defined above, designated a decision maker, identified the nearest functional comprehensive emergency obstetric care facility to use in case of emergency, identified the source of emergency funds, arranged an emergency means of transport, arranged a means of communication, and identified a suitable blood donor). Women who received antenatal care from the tertiary health facility, those with higher education, were married, who had more ANC visits, booked or were at the time of study in the third trimester and those who lived close to the health facility were more likely to prepare for birth.
Magoma 2010	Qualitative	TANZANIA, Ngorongoro district	**Study aim**: to gain an understanding of the socio-cultural and health systems factors influencing women’s decisions to seek antenatal, skilled delivery and immediate post-partum care. **Main findings**: The Maasai and Watemi women’s preferences for a home birth and lack of planning for delivery are reinforced by the failure of health care providers to consistently communicate the importance of skilled delivery and immediate post-partum care for all women during routine antenatal visits. Husbands typically serve as gatekeepers of women’s reproductive health in the two groups - including decisions about where they will deliver- yet they are rarely encouraged to attend antenatal sessions.
Magoma 2013	RCT	TANZANIA, Ngorongoro district	**Intervention:** Introduction and promotion of birth plans by care providers during ANC to prepare women and their families for birth and complication readiness. This included discussions on planned place of delivery, the importance of skilled delivery care for all women, transport arrangements to the delivery site or during an emergency, funding arrangements for delivery or emergency care services if needed, identification of possible blood donors, identification of a birth companion if desired and appropriate, and support in looking after the house- hold while the woman was at the health facility. **Main findings**: More women in the intervention arm discussed birth planning with their providers and more women delivered in health facility and attended post-natal care.
Markos 2014	Cross-sectional study	ETHIOPIA, Goba woreda, Bale Zone	**Study aim**: to assess BPCR among women of child bearing age. **Main findings:** Only 82 (14.6%) study subjects were knowledgeable about BPCR and 29% was prepared and complication ready.. Women with primary or secondary education as well as women who attended ANC were more likely to be birth prepared.
Mbalinda 2014	Mixed-Methods	UGANDA, Mulago hospital	**Study aim:** to explore the association between knowledge of obstetric danger signs and BPCR among women admitted in pregnancy with obstetric complications. **Main findings**: Only about 1 in 3 women were able to mention at least three of the five basic components of BPCR, and could be regarded as ‘knowledgeable on BPCR’. One in every 4 women could not mention any of the five components.
McPherson et al., 2006 ^a^	One group before and after evaluation	NEPAL, Siraha rural district	Community health workers (CHWs) used a BPCR package with flip charts and distributed key chains to pregnant women containing BPCR messages through monthly discussions in women’s groups. Facility-based CHWs counselled women who used facility-based services.
McPherson 2010	Process evaluation	NEPAL, Banke and Jhapa district	**Intervention:** *Jeevan Suraksha* “provides information about recommended actions to be taken at each stage of normal pregnancy and birth, identifies the danger signs that indicate possible complications, and encourages financial planning for normal births and for possible emergencies”. Content was integrated into a set of activities includ- ing 1) health education and counseling with pregnant women and household decision-makers during the antenatal period, primarily by Female Community Health Volunteers (FCHVs); (2) strengthening existing health services; and 3) postpartum home visits by FCHVs. A key aspect of the intervention was the distribution of a pictorial booklet that promotes key MNH practices to all pregnant women who are registered with FCHVs. **Main findings:** FCHVs increased their workload and expanded their role and relationship with the community. The booklet is shared and discussed among household and community members through a number of channels and clearly informs and influences household practices and decision-making. ‘Too may messages’ caused by redundancies reduces the potential impact of the booklets. Inclusion of additional household members as potential decision makers would increase effectiveness.
Midhet et al., 2010 ^a^	Cluster-RCT	PAKISTAN, rural, Khuzdar district, Balochistan province	Women and their husbands received pictorial booklets and audio cassettes, training of birth attendants in early recognition of obstetric danger signs, and providing telecommunication and transportation services for women in need of emergency obstetric and neonatal care. The intervention group consisted of a woman’s only and a couples group. Additionally, TBAs were trained for clean home delivery and owners of local vehicles were trained for referral. Healthcare providers in intervention and control arms received clinical training
Moran et al., 2006; Baya et al., 2004 ^a^	One group before and after evaluation	BURKINA FASO, Koupela	Community and facility-based HCWs and SBAs provided one-on-one counselling with pregnant women and families on key messages focused on BPCR using a flip chart. These messages were reinforced through district-based radio messages and theatre plays. Facilities were upgraded and HCWs were provided with additional training.
Mukhopadhyay 2013	Cross-sectional survey	INDIA, West Bengal, Uttar Dinajpur district	**Study aim:** to find out the perceptions and practices regarding BPCR at individual level and the related factors among pregnant and recently delivered women. **Main findings**: Half of the respondents planned for first ANC within 12 weeks, four or more ANCs and FB. Awareness of danger signs was poor with half of the women knowing at least one. Overall BPCR index of the study population was 34.5. Less than half of the women saved money and identified transport, the majority was aware of the government finance scheme.
Mullany et al., 2007 ^a^	RCT;	NEPAL, Kathmandu	Intervention group consisted of couples and women alone who received health education (two sessions) provided by health educators. The control group received no education, only a brief flyer designed to resemble and standardize the health education of normal care provided.
Mushi et al., 2010 ^a^	One group before and after evaluation	TANZANIA, rural, Mtwara region	Training of safe motherhood promoters to educate and raise awareness on maternal health aspects for pregnant women, husbands and community members through home visits. Training of safe motherhood promoters and education interventions at home and in community for pregnant women, their husbands and key community members.
Nawal 2013	DHS survey	NEPAL	**Study aim**: to assess the birth preparedness and its association with institutional delivery and postnatal check-up. **Main findings:** Only 10% of the population was well prepared for delivery. If prepared there was a greater likelihood of FB. The level of BP/CR is greater among women with pregnancy complications, lower age group, and higher education and economic status and with greater women autonomy. **Note:** The MOHP implemented the birth preparedness package. The guidelines recommend that families should save money for emergencies, arrange transportation in advance based on local conditions, identify persons who can and are eligible to donate blood if required, identify and contact health facilities and health workers who can provide services, and have a clean delivery kit
Pasha 2013	Cluster-RCT	MULTI-COUNTRY	**Intervention**: Cluster teams of trainers were formed who facilitated a multi-faceted intervention including: 1) Community mobilization to establish village-level core groups and to strengthen community capacity to identify and address barriers to obstetric and neonatal care, 2) Home-Based Life Saving Skills (HBLSS) for birth attendants and families, 3) Improvement of quality of care in existing health facilities through a combination of facility staff Emergency Obstetric and Newborn Care (EMONC) training and health facility audits. Community mobilization and birth attendant training focused on birth planning and transportation to a hospital; **Main findings:** No differences were seen between control and intervention group on primary and secondary outcomes.
Sinha et al., 2008 ^a^	One group before after	INDIA, rural, Andhra Pradesh, Rangareddy district	The intervention involved awareness raising and community support for pregnant women through local government and youth committees; involvement of their families (particularly husbands) in pregnancy-related care through monthly meetings; and bi-monthly home visits by a community organizer who worked with families to create a birth preparedness plan and support access to care
Skinner et al., 2009 ^a^	Qualitative	CAMBODIA, Kampong Chhnang	Dissemination and discussion of visual aids on danger signs and BPCR with families and communities.
Stanton 2004	Literature review	N/A	**Study aim:** To provide a detailed discussion of the issues involved in measuring birth preparedness in support of safe motherhood with the aim of improving the design and conduct of future research. **Main findings:** For effective BPCR evaluations there is a greater demand for data from or about women who are currently pregnant and conventional sampling approaches will not generate a sufficient sample of current pregnant women and recently delivered women. Additionally documenting birth preparedness requires data on knowledge, intentions, and behaviors associated with live and stillbirths.
Sood et al., 2004 ^a^	One group before and after evaluation	NEPAL, rural, districts of Bagalung and Lalitpur	Social mobilization campaigns. The Suami SIAGA (alert husband) campaign delivered through the following mass media campaigns: The Warga SIAGA encouraged community members to be alert and prepared for births within the community. The Bidan SIAGA promoted the midwife as preferred maternal healthcare provider. The Desa SIAGA campaign encouraged villages to establish lifesaving systems for women with obstetric emergencies.
Sood et al., 2004 ^a^	Pre and post comparative study with a control group	INDONESIA, West Java	Encourage and promote birth preparedness on each level directly targeting husbands, villages and communities through several (media) campaigns. In addition midwives received skills training both clinically as in communicating the basics of BPCR to their clients during ANC.
Soubeiga, 2013	Retrospective cohort study	BURKINA FASO, Koupela and Dori district	**Study aim:** to examine whether BPCR counseling provided to during routine prenatal visits increased the probability of delivering in a health facility. **Main findings:** Exposure to information varied and not all BPCR messages were received equally. The four messages together (information on danger signs; promotion of facility-based delivery; information on the cost of delivery and advice on transportation during labour and in cases of obstetric emergencies) did not significantly influence the use of SBA.
Soubeiga, 2014	Systematic Review	N/A	**Study aim**: to evaluate the impact BPCR interventions in reducing maternal and neonatal mortality in low-income countries. **Main findings**: Meta-analysis showed no significant reduction on maternal mortality but identified an 18% reduction in neonatal mortality risk. There was a slight increase in the probability of facility-delivery. **Note**: seven out of the twelve included studies implemented action-learning cycles with women’s groups.
Taleb 2015	Qualitative program evaluation	BANGLADESH, Netrokona district	**Intervention:** Implementation of Individuals, Families and Communities (IFC) program which focused on BPCR and working with TBAs to serve a new role in MNH which prioritized education, referral and social support of women rather than birth attendance with the aim to influence the social and cultural norms and practices surrounding care seeking in order to increase the utilization of skilled care. In order to promote BPCR, community- and facility-based health workers were trained to assist pregnant women and their families in creating a plan and to build community awareness of the importance of BPCR. A Birth and Emergency Preparedness Plan (BEPP) card was produced illustrating the following preparations: selecting a birth attendant; choosing a birth place and transportation to reach the birthplace; organizing with a birth companion; identifying a potential blood donor; developing a strategy to save money for costs related to pregnancy; and identifying where to seek care in the case of complications. Women receive the card either from health care providers in facilities during ANC visits or from CHWs through home visits. **Main findings:** Qualitative assessment revealed a more general trend towards planning for birth and complications and increase in knowledge of danger signs. Additionally a shift was identified in choosing for skilled care, although this was primarily for choosing community-based skilled birth attendant (CBSBA) at home.
Turan et al., 2011^a^	Quasi-experimental pre and post study with non-equivalent control group	ERITREA, one district in the Red zone and another in the Anseba zone	Training of community members (women and men) to become maternal health volunteers and lead participatory education sessions making use of materials developed. Skills training for health care providers was also conducted.
Turan 2014	Prospective cohort study	ETHIOPIA, Jimma Zone	**Study aim:** to determine the effect of BPCR on skilled care. One third of the respondents gave birth with skilled care. **Main findings:** Two thirds of women who planned SBA actual used it. Reasons for non-use of SBA despite planning were: labor was not associated with problems, lack of transport, lack of money for transport and services. Women who had knowledge of key danger signs and were well prepared (performing three or more BPCR actions: planed to save money, planed to arrange transport, planed to give birth in health facility, planed to be attended by skilled attendant and planed to arrange blood donor) were more likely to use SBA.
Urassa 2012	Cross-sectional survey	TANZANIA, Mpwapwa District	**Study aim:** To assess knowledge and practices with respect to BPCR amongst women who recently delivered in Mpwapa District. **Main findings:** The majority of the women had decisions made on place of delivery, a person to make final decision, a person to assist during delivery, someone to take care of the family, a person to escort her to health facility and planned to be delivered by a SBA. Age of the woman, education level, marital status, number of ANC visits and knowing ≥3 obstetric danger signs were associated with birth preparedness and complication readiness.

**Table 2:** BPCR implementation strategies employed by included studies

Type of strategy	Studies employing the strategy
House visits by volunteers who provide BPCR education	Ahluwalia et al. 2003; Baqui 2008; Darmstadt et al. 2010; Dickerson 2010; Hodgins et al. 2009; Hossain and Ross 2006; Kumar et al. 2012b; McPherson et al. 2006; McPherson 2010; Moran et al. 2006; Mushi, Mpembeni, and Jahn 2010a; Sinha 2008; Skinner and Rathavy 2009; Taleb 2015
Training of facility healthcare workers to provide BPCR education as part of antenatal care	Brazier et al. 2009; Dickerson 2010; Family care international 2007a, 2007b; Fonseca-Becker and Schenck-Yglesias 2004; Jennings 2010; Moran et al. 2006; Mullany, Becker, and Hindin 2007; Soubeiga, 2013; Sood, Urvashi, Palmer, et al. 2004; Taleb 2015
Providing women (and families) with booklets/flipcharts or other visual aids with BPCR information	Brazier et al. 2009; Family care international 2007a, 2007b; Fonseca-Becker and Schenck-Yglesias 2004; Hodgins et al. 2009; Hossain and Ross 2006; McPherson et al. 2006; McPherson 2010; Midhet and Becker 2010; Moran et al. 2006; Skinner and Rathavy 2009; Sood, Urvashi, Mishra, et al. 2004); Taleb 2015
Community mobilization activities (Participatory groups, village meetings or community theater and songs with BPCR information)	Brazier et al. 2009; Family care international 2007a, 2007b; Fonseca-Becker and Schenck-Yglesias 2004; Hossain and Ross 2006; Midhet and Becker 2010; Moran et al. 2006; Mushi, Mpembeni, and Jahn 2010a; Pasha 2013; Sinha 2008; Skinner and Rathavy 2009; Sood, Urvashi, Mishra, et al. 2004; Sood, Urvashi, Palmer, et al. 2004; Turan, Tesfagiorghis, and Polan 2011)
Media campaign (radio, jingles, posters, leaflets) with BPCR messages	Fonseca-Becker and Schenck-Yglesias 2004; Moran et al. 2006; Sood, Urvashi, Mishra, et al. 2004; Sood, Urvashi, Palmer, et al. 2004)
Improving (quality of) BPCR of health facilities	Pasha 2013;
No strategy, descriptive studies	Acharya et al. 2015; Agarwal 2010; August 2015; Choudhury 2011; Debelew 2014; Ekabua 2011; Hailu 2011; Hiluf 2007; Iliyasu 2010; Kabakyenga 2011; Kabakyenga 2012; Kakaire 2011; Kaso 2014; Kuteyi 2011; Magoma 2010; Magoma 2013; Markos 2014; Mbalinda 2014; Mukhopadhyay 2013; Nawal 2013; Tura 2014; Urassa 2012
No strategy, literature review	Stanton 2004; Soubeiga 2014

Studies define BPCR and its main components variously which complicates interpretation of results, context and policy advice. For example, the most commonly described components include: identifying funds for birth and emergency expenses; deciding on a preferred birth attendant; identifying transport to the health facility for birth or complications; choosing the place of birth and location of nearest facility; and knowledge and identification of danger signs in pregnancy. Other definitions include identifying compatible blood donors, preparing supplies, identifying a birth companion, and discussing plans with husband and family. In the systematic review, it was impossible to determine which strategy and which components, or which combination of strategies and components, was most effective in improving health seeking outcomes [1]. Despite these differences, this analysis of contextual and implementation factors provides an understanding of some common barriers and facilitators to implementing interventions that promote BPCR. The SURE framework is added as Additional file 1.

### Stakeholder perspectives on BPCR

All studies involved women and their families; some specifically addressed communities at large [42–45]; and three studies specifically targeted health care workers [43, 46, 47]. Descriptive studies of BPCR almost exclusively evaluated preparedness of women [9, 11, 13, 16, 17, 19, 21, 27, 29, 30, 35, 48], with the exception of Iliyasu et al. (2010) and August et al. (2013) that specifically assessed BPCR among husbands [18, 26]. The JHPIEGO Maternal and Newborn Health Program and Skilled Care Initiative directed BPCR interventions at individuals, communities, facilities and policy level [49, 52].

#### Perspectives of women and their families

Some studies reported women’s perceptions of birth as a normal and ‘natural event’ which could be successful at home, and that this often reduced the urgency to plan for facility birth. Other studies similarly reported beliefs that pregnancy outcomes are predetermined and ‘in God’s hands’, therefore there was no perceived need to be prepared for birth [13, 14, 45, 63]. In two studies in Tanzania and Nepal ‘modern’ health services were regarded as the ‘last resort’ to be used only after complications arose [14, 55]. Improving knowledge of danger signs is an essential element of most BPCR interventions. However, promoting this knowledge without ensuring awareness of the need for planning for normal birth might unintentionally results in the perception that no actions need to be taken if all signs are ‘absent’ and promote the notion that uncomplicated births indeed are best at home [14]. Quasi-experimental studies identified strong correlations between education level and BPCR, and concluded that BPCR interventions were more successful, and facility delivery more likely, among women with higher levels of education [11, 13, 15, 17, 21, 56, 57].

We found evidence in the studies that despite being able to recognise danger signs during pregnancy sometimes women remain silent and do not seek care because of cultural beliefs about the underlying causes. For example, in Tanzania obstructed labour, retained placenta and eclampsia were associated with adultery [47, 58]. In some countries BPCR actions are limited due to fear of unfavourable outcomes and the belief that ‘preparing’ could bring bad luck [20, 45]. In Tanzania and Kenya, although families reportedly discussed pregnancy and childbirth together (including husbands and wives), the studies indicate that taboos still exist and that this can restrict BPCR discussions. For example, announcing pregnancy and informing the husband when labour starts, is believed to bring misfortune [53], limiting husbands’ ability to make timely preparations [47]. Cultural beliefs and norms also hindered transport preparations in some contexts, as women refrained from crossing a river since this was believed to cause abortion/preterm birth [47], and travel at night was considered dangerous due to active evil spirits [20]. In Bangladesh and Kenya, purchasing relevant items in preparation for birth was reportedly discouraged, especially items for the baby. Financial preparations were perceived as wasteful as it is unknown if the child would survive [20, 53, 58].

Identifying a SBA beforehand was a key BPCR message in all studies, and this inevitably involved making a choice about where to give birth and with whom. Some studies emphasized the importance of providing women with clear information during ANC on who is considered a SBA [50, 55]. In some contexts, traditional birth attendants (TBAs) are the preferred attendant as the first point of care in pregnancy and when complications arise. They are considered ‘skilled’ because of their years of experience, ability to perform important rituals and willingness to attend women at home [48, 59, 60]. Similarly, women’s understanding of the expected date of birth could limit timely preparations for facility birth or birth with a SBA, as some women perceived the estimated date of birth as exact date of birth, thus awaiting this exact time to make further plans [14, 20, 45, 47].

Most interventions took place in contexts where men or other family members are the main decision makers and gatekeepers to women’s timely access to care. Despite this, men are often excluded from maternal health interventions, and this can impact on the likelihood that BPCR actions are taken. In studies in Tanzania women reported that men may cause delay in seeking transport for women in labour or with complications for several reasons: they are unavailable at the time, they may not be aware of the emergency, or they feel ashamed to be seen supporting their wives [14, 47]. A study in India reported that although men appear willing to perform certain tasks, primarily related to financial contributions, they often do not take on more proactive or supportive roles and are reluctant to get fully involved [61]. In a Nepali programme, women requested volunteers to increase awareness of husbands and mothers in-law, to help them in childbirth preparations [55]. Descriptive studies indicate that when men are involved in making plans for birth, they more often accompany their wives to the antenatal clinic and labour ward [16, 18, 45]. Some studies specifically involved men, by providing information to men (68), or training men as maternal health promoters (56, 58). In the latter intervention, implemented in Eritrea and Tanzania, men delivered BPCR messages to households and communities, which was well received and contributed to men’s understanding of the importance of timely care seeking during pregnancy and for childbirth.

#### Perspectives of community stakeholders

Pregnancy and childbirth are usually regarded as family events and the wider community rarely plays a major role in preparation or readiness activities [47, 50, 61]. However, other community stakeholders who were not always targeted by BPCR interventions, such as community leaders, responded positively to interventions and implementers [57, 61, 62]. Interventions that did include community BPCR components resulted in increased awareness of maternal deaths in the community [43] and increased feeling of responsibility for pregnant women in the community. The latter through interventions which were specifically applicable to communities at large [50], such as developing transport or financial support systems, or through linking interventions to existing community structures [43, 47]. In one study in Nepal, women requested more detailed information on where to go for birth and how to arrange transport [55].

In one study in India, community leadership was particularly supportive where youth groups held activities to increase awareness of maternal health problems, which subsequently increased direct interaction with government officials on problems faced by health providers and the women themselves [61]. Community transport and financial schemes for maternal emergencies were successful if supervised by transparent, trustworthy and stable leadership [57, 63] and reversely inefficient when corrupted or insufficiently managed [43, 57].

Implementers of BPCR messages, health workers or volunteers, were generally well accepted by individuals, families and communities. In one Tanzanian study, home visits by volunteers were especially appreciated for the time spent on discussions and questions [45]. BPCR messages were easily understood by women respondents in Nepal and Burkina Faso [23, 52]. Studies that used visual aids such as cards, posters or booklets were positively received and understood [32, 42, 43, 45, 50].

#### Perspectives of health workers and BPCR implementers

Although most studies trained facility-based health workers to implement BPCR interventions either at facility or in the community [42, 43, 49, 51–53, 55, 56, 64–66], other studies worked mainly with community health workers [47, 50, 52, 55, 56, 59, 67] including TBAs [43, 50] and community volunteers [42, 44, 45, 61, 64, 65, 68, 69]. In studies in Tanzania, facility-based health workers indicated they felt appreciated by the community volunteers, which increased collaboration [45, 47]. In another Tanzanian study, TBAs changed from childbirth care providers to educators, counsellors and referral advisors, thus becoming active promoters of skilled attendance at birth [45].

Implementers generally reported they were satisfied with their activities and job aids [22, 23, 42, 45, 47] and felt supported by combinations of job aids with training, field support, and organizational change [22, 23]. Some village volunteers felt appreciated by their communities and were committed to activities [47, 55], others felt overburdened by study tasks [23]. Implementers in Nepal and Benin reported that job aids included too many or a repetition of messages or lacked concrete activities or examples for preparations to effectively help women and their families [22, 23]. Facility interventions of BPCR during ANC in Benin and Tanzania increased workload by requiring more time with pregnant women [14, 22].

### Health service delivery factors

In some studies hospital staff and researchers noted that shortage of staff at facilities, in particular during nights and weekends, and high staff turnover limited intervention effectiveness. For example high turnover of facility personnel in Tanzania and Bangladesh [43, 56], limited government training or supervision of staff in Burkina Faso and India [64, 70] and limiting staff payments, causing health staff to run private business and as a consequence SBA absence in the clinics in Cambodia [42]. Giving staff additional tasks while at the same time maintaining morale and commitment was not easy, despite provision of additional training [43, 47, 49, 51, 53, 56, 64, 66]. Several BPCR interventions reported in the included studies incorporated service delivery improvements including training of facility-based [43–45, 48, 49, 52, 54, 68] and community-based [46, 47, 64, 67] health workers on (emergency) obstetric care, improving facility infrastructure and supplies [43, 47, 53, 56, 64] as well as strengthening the transport and referral system [46, 51, 53, 56, 64]. A study in Tanzania made additional effort to improve the supply of drugs, provided essential obstetric equipment, and facilitated strengthening of the logistics system at facility level [56]. However, no improvements were seen as ordered supplies were frequently not available mainly due to logistics problems at higher levels in the health system [56].

Women’s and family member’s views about the accessibility of care seemed to influence their willingness to prepare for facility birth. Lack of availability of care 24 h a day [42] or lack of awareness that lower-level health facilities also provide childbirth services [53] could result in women choosing home birth. Even if women considered facility birth, large distances to nearest facilities, poor road conditions and lack of transport options [47, 52–54] made facility birth unrealistic. Cost for transportation, formal and informal costs for facility-based services and general out-of-pocket payments limited affordability of care [14, 47, 53, 55, 56, 61] and made TBA care often the cheaper option [42]. Perceived lack of quality of care at facilities including accounts of staff being described as inattentive and unconcerned or needing bribes before treatment was another barrier for SBA [53, 58].

### Social and political factors

Three studies in Nepal show implementation was affected by political instability and civil war, hindering implementation location or intensity [50, 55, 68]. The situation in Nepal reduced accessibility to health facilities due to security concerns in some areas [68] and in one study changed the implementation location from rural to urban although the intervention was thought to be most effective in rural geographical locations [50].

BPCR interventions in the studies included in this review were primarily implemented in patriarchal societies where gender inequality pervades household decision making [54, 57, 71]. Several interventions attempted to address this specific barrier to BPCR, by involving locally influential people. For example, TBAs, traditional healers, church leaders, community or clan elders and political leaders were consulted prior to the intervention or were encouraged to become involved as active promoters [62, 63, 66]. In Indonesia, promotion material featured a popular singer which helped to give the campaign a brand name which spoke to the majority of the target population [49].

One study in Tanzania showed benefits of collaboration with key-stakeholders and government leadership [47], in other countries government policies or changes in for example payment regulations could preclude BPCR implementation [42]. Although national policy changes in favour of MNHC priorities assisted the implementation process in Guatemala [51], BPCR implementation was limited by other national policy changes, such as ending cost-sharing policy in Kenya, increasing overall costs of care [53], and consequently reports of corruption [53, 56].

Good rapport between the donor agency and government workers together with communities facilitated BPCR implementation, especially when engaging communities in problem solving in relation to BPCR [57, 68, 71]. Translating Non Governmental Organization (NGO) concepts into locally meaningful capacities or strategies, not only required time, but also adaptive skills from NGO workers from headquarter to local NGO level in Kenya and Tanzania [48, 58]. Ability of national and local policy makers to continuously connect with communities and hospitals, through newly established village health worker committees or supportive supervision of health services, seem important factors for sustainability of interventions and finances [51, 63]. Scale-up of the BPCR intervention was facilitated in Burkina Faso by connecting to existing health system structures [71]. In Cambodia, short-term BPCR interventions implemented independently of the health system structure, led to fear among programme planners and implementers, that project staff would become unemployed or move to the private sector once the intervention ended [42].

## Discussion

### Linking implementation factors with improvements in care seeking outcomes

For studies included in the original systematic review that reported improvements in use of SBA or birth in a facility, the important factors that seemed to make a difference were positive perceptions of the intervention among women, families and community members and readily understood BPCR messages which motivated behaviour change [43, 45, 52]. Positive behaviour change, which could be making more preparations for childbirth or choosing to give birth with a skilled attendant, seemed to be more likely in women with higher levels of formal education [11, 13, 15, 17, 21, 56, 57], when husbands (as well as women) were targeted with BPCR messages on the need for SBA [43, 45, 52, 61], or when the intervention helped to lessen the influence of traditional or cultural barriers [51]. In studies where BPCR interventions led to no or marginal improvements in number of women giving birth with a skilled attendant or at a facility, preference for home birth and underlying cultural beliefs mitigated the need for facility birth [47, 53, 55].

Political instability [50, 55, 68] and short duration of interventions [42, 50] were reported to be among factors which prevented BPCR interventions impacting on care seeking outcomes. On the contrary, in studies reporting improvements in SBA or facility birth, BPCR was generally implemented within a package of interventions designed to address both demand and supply strengthening [43, 46, 49, 51, 56, 64]. It is well recognized that community sensitization to BPCR without concurrent improvements in access to facilities and the quality of care provided will have little impact on care seeking or other health outcomes [43, 55]*.* When interventions were integrated into existing government health service delivery systems [51, 70], or were delivered in partnership with relevant safe motherhood stakeholders to ensure close links between the community and facilities [43, 45], this seemed to increase impact on care-seeking outcomes. According to study evaluations the inclusion of perceptions of hospital management and sub-district policy makers on BPCR should be part of and could facilitate NGO implementation strategies [65]. These stakeholders could be offered support to make quality improvements in health policy making [72].

### Limitations

Firstly, the variety of definitions and topics used to describe BPCR complicate interpretation of results, context and policy advice. Seven authors did not specify any BPCR definition [42, 46, 53, 56, 61, 64, 73]. Six interventions were focused on complication readiness only [43, 46, 47, 59, 67, 70]. Five authors did explain that BPCR should also include preparedness for routine birth, [48, 51–54] although in one study this was not part of the intervention [43]. In areas with extremely low SBA use, ensuring improved conditions at home [preparing birth kits, a clean confinement room] were considered improvements in BPCR [43, 46, 47, 59, 67]. Secondly, skilled birth attendants are variously described in the included studies, making extrapolation of results from one context to another difficult. Ten studies defined skilled birth attendants as [47, 51–53, 55, 56, 59, 64, 67, 70] doctors, midwives or nurses. In some studies - as per WHO definition - unqualified staff including nurse aides were considered SBAs [47, 53, 56, 64]. Other studies reported on health facility births [43, 44, 46, 49, 51, 61, 64, 67, 68] which does not necessarily mean the presence of a SBA. Two interventions included promotion of skilled care irrespective of location which consequently resulted in inclusion of home birth with SBAs [45, 50]. As study contexts varied vastly, comparing studies on intervention and outcomes remains difficult. In our view defining interventions and outcome measures properly is crucial in BPCR, as improper definitions complicate interpretation of outcomes.

Further research of robust design is needed to: agree on key definitions and priority BPCR actions; assess the effect of including men and other key-stakeholders on care-seeking outcomes; and to understand how cultural factors influence BPCR implementation [5]. Although we aimed to include additional qualitative studies, only few were identified. Few studies reported on barriers or facilitators related to the intervention or program itself, such as resource implications, intervention integrity, leadership, and only some reported on donor policies or legislations. Often studies did not elaborate how and why such factors lead to successful or non-successful outcomes. These aspects also require further consideration in future research on BPCR interventions or packages.

The SURE framework proved useful in assessment of factors influencing implementation although we would argue that especially in low-resource settings ‘cultural factors’ should be added as a separate category. Cultural factors are generally under researched in maternal health studies [74] and such adaptation of existing models could form a way to swiftly create insight into complexities of implementing health interventions locally [75].

## Conclusion

Implementation of BPCR interventions to improve the use of skilled care at birth requires careful consideration of contextual factors influencing implementation. When developing programmes and interventions, BPCR messages and strategies should match and respect the target audience and the different decision makers in maternal health and their values, as well as the organisation and capacities of the local health system. When mismatch occurs, such as when increased demand for facility births meets unprepared facilities in contexts where essential and comprehensive emergency obstetric care services are not available, this could cause considerable damage to the often already fragile trust the community has in the formal health system and increase complications or mistreatment of women giving birth at facilities. For this reason, it is important that BPCR is implemented alongside other interventions and activities to strengthen the supply and improve the quality of maternity care services. Implementation of BPCR should always include preparedness for both routine childbirth care and for complications, ensuring women and families have discussed the plans. Local socio-economic realities and determinants however remain a heavy burden for effective implementation of BPCR, and therefore require actions with the community and other stakeholders. BPCR messages should therefore be adapted to the local context in terms of availability, accessibility and affordability of health facilities and services. Inclusive and active involvement of all levels of stakeholders, including health officials and policymakers, appears to be a crucial step for securing linkages between the actions of all respective stakeholders that optimizes chances for women and newborns to reach needed care and contributes to the success of BPCR.

## Additional file

Additional file 1: Checklist for identifying factors affecting the implementation of a policy option (DOCX 99 kb)

## Abbreviations

ANC: Antenatal care
BPCR: Birth Preparedness and Complication Readiness
JHPIEGO: Johns Hopkins Program for International Education in Gynecology and Obstetrics NGO Non Governmental Organization
SBA: Skilled Birth Attendant
SURE: Supporting the Use of Research Evidence
TBA: Traditional Birth Attendant
WHO: World Health Organization

## References

[1] Solnes Miltenburg A, Roggeveen Y, Shields L, van Elteren M, van Roosmalen J, Stekelenburg J, Portela A (2015). Impact of birth preparedness and complication readiness interventions on birth with a skilled attendant: a systematic review. PlosOne.

[2] JHPIEGO/Maternal and Neonatal Health (MNH) Program. Birth Preparedness and Complication Readiness: A Matrix of Shared Responsibility. Baltimore, MD; 2001.

[3] Carroli G, Villar J, Piaggio G, Khan-Neelofur D, Gülmezoglu M, Mugford M (2001). WHO systematic review of randomised controlled trials of routine antenatal care. Lancet.

[4] Di Mario S, Basevi V, Gori G, Spettoli D (2005). What is the effectiveness of antenatal care? (supplement).

[5] World Health Organization. WHO recommendations on health promotion interventions for maternal and newborn health 2015. 2015. 26180864

[6] Solnes Miltenburg A, Roggeveen Y, van Elteren M, Shields L, Bunders J, van Roosmalen J (2013). A protocol for a systematic review of birth preparedness and complication readiness programs. Syst Rev.

[7] The mapping “Health system and community-based interventions for improving maternal health and for reducing maternal health inequalities in low- and middle-income countries: a two-stage mixed-methods research synthesis” was conducted under the European Union “Multilateral Association for Studying Health inequalities and enhancing north-south and south-south Cooperation” (MASCOT) project and the MH-SAR project (Maternal Health South Africa-Rwanda funded by the Netherlands Organization for Scientific Research. The WHO provided technical and financial support during the mapping for reviewers to identify articles addressing health promotion interventions for maternal health, including birth preparedness and complications readiness.

[8] Kabakyenga JK, Ostergren P-O, Turyakira E, Pettersson KO (2012). Influence of birth preparedness, decision-making on location of birth and assistance by skilled birth attendants among women in south-western Uganda. PLoS One.

[9] Urassa DP, Pembe AB, Mganga F (2012). Birth preparedness and complication readiness among women in Mpwapwa district, Tanzania. Tanzan J Health Res.

[10] Agarwal S, Sethi V, Srivastava K, Jha PK, Baqui AH (2010). Birth preparedness and complication readiness among slum women in Indore city, India. J Health Popul Nutr.

[11] Hiluf M, Fantahun M (2007). Birth preparedness and complication readiness among women in Adigrat town, north Ethiopia. Ethiop J Heal Dev.

[12] Dickerson T, Simonsen S, Samen A (2010). Pregnancy and village outreach Tibet a descriptive report of a community- and home-based maternal-newborn outreach program in rural Tibet. J Perinat Neonat Nurs.

[13] Mukhopadhyay DK, Mukhopadhyay S, Bhattacharjee S, Nayak S, Biswas AK, Biswas AB (2013). Status of birth preparedness and complication readiness in Uttar Dinajpur District, West Bengal. Indian J Public Health.

[14] Magoma M, Requejo J, Campbell OMR, Cousens S, Filippi V (2010). High ANC coverage and low skilled attendance in a rural Tanzanian district: a case for implementing a birth plan intervention. BMC Pregnancy & Childbirth.

[15] Kuteyi EAA, Kuku JO, Lateef IC, Ogundipe JA, Mogbeyteren T, Banjo MA (2011). Birth preparedness and complication readiness of pregnant women attending the three levels of health facilities in Ife central local government, Nigeria. Journal of Community Medicine & Primary Health.

[16] Kakaire O, Kaye DK, Osinde MO (2011). Male involvement in birth preparedness and complication readiness for emergency obstetric referrals in rural Uganda. Reprod Health.

[17] Kabakyenga JK, Östergren P-O, Turyakira E, Pettersson KO (2011). Knowledge of obstetric danger signs and birth preparedness practices among women in rural Uganda. Reprod Health.

[18] Iliyasu Z, Abubakar IS, Galadanci HS, Aliyu MH (2010). Birth preparedness, complication readiness and fathers’ participation in maternity care in a northern Nigerian community. Afr J Reprod Health.

[19] Ekabua JE, Ekabua KJ, Odusolu P, Agan TU, Iklaki CU, Etokidem AJ (2011). Awareness of birth preparedness and complication readiness in southeastern Nigeria. ISRN Obstet Gynecol.

[20] Choudhury N, Ahmed SM (2011). Maternal care practices among the ultra poor households in rural Bangladesh: a qualitative exploratory study. BMC Pregnancy Childbirth.

[21] Hailu M, Gebremariam A, Alemseged F, Deribe K (2011). Birth preparedness and complication readiness among pregnant women in southern Ethiopia. PLoS One.

[22] Jennings L, Yebadokpo AS, Affo J, Agbogbe M (2010). Antenatal counseling in maternal and newborn care: use of job aids to improve health worker performance and maternal understanding in Benin. BMC Pregnancy Childbirth.

[23] McPherson R, Tamang J, Hodgins S, Pathak LR, Silwal RC, Baqui AH (2010). Process evaluation of a community-based intervention promoting multiple maternal and neonatal care practices in rural Nepal. BMC Pregnancy Childbirth.

[24] Stanton CK (2004). Methodological issues in the measurement of birth preparedness in support of safe motherhood. Eval Rev.

[25] Magoma M, Requejo J, Campbell O, Cousens S, Merialdi M, Filippi V (2013). The effectiveness of birth plans in increasing use of skilled care at delivery and postnatal care in rural Tanzania : a cluster randomised trial. Tropical Med Int Health.

[26] August F, Pembe AB, Mpembeni R, Axemo P, Darj E. Men’s knowledge of obstetric danger signs. Birth Preparedness and Complication Readiness in Rural Tanzania. 2015:1–12. 10.1371/journal.pone.0125978 PMC4423869 25950814

[27] Kaso M, Addisse M. Birth preparedness and complication readiness in robe Woreda, Arsi Zone, Oromia Region, Central Ethiopia: a cross-sectional study 2014;11[1]:1–12. 10.1186/1742-4755-11-55 PMC4118259 25038820

[28] Karkee R, Lee AH, Binns CW (2013). Birth preparedness and skilled attendance at birth in Nepal : implications for achieving millennium development goal 5. Midwifery.

[29] Markos D, Bogale D (2014). Birth preparedness and complication readiness among women of child bearing age group in Goba woreda, Oromia region, Ethiopia. BMC Pregnancy Childbirth.

[30] Nawal D, Goli S (2013). Birth preparedness and its effect on place of delivery and post-natal check-ups in Nepal. PLoS One.

[31] Pasha O, McClure EM, Wright LL, Saleem S, Goudar SS, Chomba E (2013). A combined community- and facility-based approach to improve pregnancy outcomes in low-resource settings: a global network cluster randomized trial. BMC Med.

[32] Taleb F, Perkins J, Ali NA, Capello C, Ali M, Santarelli C (2015). Transforming maternal and newborn health social norms and practices to increase utilization of health services in rural Bangladesh: a qualitative review. BMC Pregnancy Childbirth.

[33] Tura G, Afework MF, Yalew AW. The effect of birth preparedness and complication readiness on skilled care use : a prospective follow-up study in Southwest Ethiopia. Reprod Health 2014;11[1]:1–10. 10.1186/1742-4755-11-60 PMC4127036 25091203

[34] Mbalinda SN, Nakimuli A, Kakaire O, Osinde MO, Kakande N, Kaye DK (2014). Does knowledge of danger signs of pregnancy predict birth preparedness ? A critique of the evidence from women admitted with pregnancy complications. Health Res Policy Syst.

[35] Debelew G, Afework M, Yalew AW (2014). Factors affecting birth preparedness and complication readiness in Jimma zone. Southwest Ethiopia: a multilevel analysis.

[36] Acharya A, Kaur R, Prasuna J, Rasheed N (2015). Making pregnancy safer—birth preparedness and complication readiness study among antenatal women attendees of a primary health center, Delhi. Indian J Community Med.

[37] Soubeiga D, Gauvin L, Hatem M (2014). A, Johri M. Birth preparedness and complication readiness [BPCR] interventions to reduce maternal and neonatal mortality in developing countries: systematic review and meta-analysis. BMC Pregnancy Childbirth.

[38] Soubeiga D, Sia D, Gauvin L. Increasing institutional deliveries among antenatal clients: effect of birth preparedness counselling. Health Policy Plan. 013:1–10. 10.1093/heapol/czt089 PMC4256950 24270519

[39] The SURE Collaboration. SURE Guides for Preparing and Using Evidence-Based Policy Briefs: identifying and addressing barriers to implementing policy options.. 2011. Available from: http://global.evipnet.org/SURE-Guides/

[40] Glenton C, Colvin CJ, Carlsen B, Swartz A, Lewin S, Noyes JRA. Barriers and facilitators to the implementation of lay health worker programmes to improve access to maternal and child health: qualitative evidence synthesis. CochDatabase Syst Rev. 2013;(10) 10.1002/14651858.CD010414.pub2. 10.1002/14651858.CD010414.pub2 PMC6396344 24101553

[41] Smith HJ, Colvin CJ, Richards E, Roberson J, Sharma G, Thapa K, Gülmezoglu AM. Programmes for advance distribution of misoprostol to prevent post-partum haemorrhage: a rapid literature review of factors affecting implementation. Health Policy Plan. 2015; 10.1093/heapol/czv012 25797470

[42] Skinner J, Rathavy T (2009). Design and evaluation of a community participatory, birth preparedness project in Cambodia. Midwifery.

[43] Hossain J, Ross SR (2006). The effect of addressing demand for as well as supply of emergency obstetric care in Dinajpur, Bangladesh. IJOG.

[44] Turan J, Tesfagiorghis M, Polan M (2011). Evaluation of a community intervention for promotion of safe motherhood in eritrea. J Midwifery Womens Health.

[45] Mushi D, Mpembeni R, Jahn A (2010). Effectiveness of community based safe motherhood promoters in improving the utilization of obstetric care, The case of Mtwara Rural District in Tanzania. BMC Pregnancy and Childbirth.

[46] Midhet F, Becker S (2010). Impact of community-based interventions on maternal and neonatal health indicators: results from a community randomized trial in rural Balochistan, Pakistan. Reprod Health.

[47] Ahluwalia IB, Schmid T, Kouletio M, Kanenda O (2003). An evaluation of a community-based approach to safe motherhood in northwestern Tanzania. IJOG.

[48] Karkee R, Lee AH, Binns CW (2013). Birth preparedness and skilled attendance at birth in Nepal: implications for achieving millennium development goal 5. Midwifery.

[49] Sood S, Urvashi C, Palmer A, Molyneux I (2004). Measuring the effects of the SIAGA behavior change campaign in Indonesia with population-based survey results.

[50] Sood S, Urvashi C, Mishra P, Neupane S (2004). Measuring the effects of behavioral change intervention in Nepal with population based survey results.

[51] Fonseca-Becker F, Schenck-Yglesias C (2004). Measuring the effects of behavioral change and service delivery interventions in Guatemala with population based survey results.

[52] Moran AC, Sangli G, Dineen R, Rawlins B, Yaméogo M, Baya B (2006). Birth-preparedness for maternal Health : findings from Koupéla District. Burkina Faso.

[53] Family Care international. Testing Approaches for Increasing Skilled Care During Childbirth : Key Findings from Homabay and Migori Districts, Kenya. New York: Family Care International. 2007.

[54] Moore M, Copeland R, Chege I, Pido D, Griffiths M. A behavior Change Approach to investigating Factors Influencing Women’s Use of Skilled Care in Homa Bay District, Kenya. 2002. Washington, D.C.

[55] McPherson R A, Khadka N, Moore JM, Sharma M. Are birth-preparedness programmes effective? Results from a field trial in Siraha district, Nepal. J Health Popul Nutr 2006;24[4]:479–488. PMC3001152 17591345

[56] Family Care international. Testing Approaches for Increasing Skilled Care During Childbirth : Key Findings from Igunga District, Tanzania. New York: Family Care International. 2007.

[57] Barbey A, Faisel AJ, Myeya J, ADD M, Stavrou V, Psych MC, et al. Dinajpur SafeMother initiative final evaluation report. CARE. 2001;

[58] Kaharuza F, Mimba T, Pesa W. Birth preparedness in rural Tanzania. CARE. 2001;

[59] Kumar V, Kumar A, Das V, Srivastava NM, Baqui AH, Santosham M (2012). Community-driven impact of a newborn-focused behavioral intervention on maternal health in Shivgarh, India. Int J Gynaecol Obstet.

[60] Roggeveen Y, Birks L, van Kats J, Manyama M, Hatfield J, JFG B (2013). Low utilization of skilled birth attendants in Ngorongoro conservation area, Tanzania: a complex reality requiring action. Health.

[61] Sinha D (2008). Empowering communities to make pregnancy safer: an intervention in rural Andhra Pradesh. Health and population innovation fellowship programme working paper, no 5.

[62] Family Care International. Care-Seeking During Pregnancy, Delivery, and the Postpartum Period: A Study in Homabay and Migori Districts, Kenya. New York: Family Care International. 2003.

[63] Ahluwalia IB, Robinson D, Vallely L, Gieseker KE, Kabakama A (2010). Sustainability of community-capacity to promote safer motherhood in northwestern Tanzania: what remains?. Glob Health Promot.

[64] Brazier E, Andrzejewski C, Perkins ME, Themmen EM, Knight RJ, Bassane B (2009). Improving poor women’s access to maternity care: findings from a primary care intervention in Burkina Faso. Soc Sci Med.

[65] Baqui AH, Rosecrans AM, Williams EK, Agrawal PK, Ahmed S, Darmstadt GL (2008). NGO facilitation of a government community-based maternal and neonatal health programme in rural India: improvements in equity. Health Policy Plan.

[66] Mullany BC, Becker S, Hindin MJ (2007). The impact of including husbands in antenatal health education services on maternal health practices in urban Nepal: results from a randomized controlled trial. Health Educ Res.

[67] Darmstadt GL, Choi Y, Arifeen SE, Bari S, Rahman SM, Mannan I (2010). Evaluation of a cluster-randomized controlled trial of a package of community-based maternal and newborn interventions in Mirzapur, Bangladesh. PLoS One.

[68] Hodgins S, McPherson R, Suvedi BK, Shrestha RB, Silwal RC, Ban B (2009). Testing a scalable community-based approach to improve maternal and neonatal health in rural Nepal. J Perinatol [Internet].

[69] Kumar V, Mohanty S, Kumar A, Misra RP, Santosham M, Awasthi S (2008). Effect of community-based behaviour change management on neonatal mortality in Shivgarh, Uttar Pradesh, India: a cluster-randomised controlled trial. Lancet.

[70] Baqui A (2008). Impact of an integrated nutrition and health programme on neonatal mortality in rural northern India. Bull World Health Organ.

[71] Baya B, Sangli G, Maiga A. Measuring the effects of behavior change interventions in Burkina Faso with populatin-based survey results. Baltimore. 2004.

[72] Hounton S, Menten J, Ouédraogo M, Dubourg D, Meda N, Ronsmans C (2008). Effects of a skilled care initiative on pregnancy-related mortality in rural Burkina Faso. Tropical Med Int Health.

[73] Mpembeni RN, Killewo JZ, Leshabari MT, Massawe SN, Jahn A, Mushi D (2007). Use pattern of maternal health services and determinants of skilled care during delivery in southern Tanzania: implications for achievement of MDG-5 targets. BMC Pregnancy Childbirth.

[74] Gil-González D, Carrasco-Portiño M, Ruiz MT (2006). Knowledge gaps in scientific literature on maternal mortality: a systematic review. Bull World Health Organ.

[75] Birks L, Powell C, Thomas A, Medard E, Roggeveen Y, Hatfield J (2011). Promoting health, preserving culture: adapting RARE in the Maasai context of northern Tanzania. AIDS Care.

