# Supplementary material for: Factors influencing implementation of interventions to promote birth preparedness and complication readiness
Source: BMC Pregnancy Childbirth. 2017 Aug 31;17:270. doi: 10.1186/s12884-017-1448-8 (PMC5577754; doi:10.1186/s12884-017-1448-8)
Supplement: Additional file 1: — Checklist for identifying factors affecting the implementation of a policy option (DOCX 99 kb) [file 12884_2017_1448_MOESM1_ESM.docx]

# Annex 1. Checklist for identifying factors affecting the implementation of a policy option

Based on the SURE Guides for Preparing and Using Evidence-Based Policy Briefs. Available from [www.evipnet.org/sure](http://www.evipnet.org/sure)

| **Level** | **Barriers and enablers** | | **Extracts from papers**  (Specify if this is data from study findings, or information/opinion from background/discussion) |
| --- | --- | --- | --- |
| 1. Main Stakeholders from the community | Knowledge & skills | Women and their families may have varying degrees of knowledge about the healthcare issue or the intervention, or may not have the skills to engage with the intervention. E.g. People may be unaware that the intervention exists or how it is supposed to help them. | **[Unaware of importance of care for pregnant women] Sinah**: Discussions revealed that pregnancy was perceived as a normal event in the life of a woman, and one in which the community did not have a role to play. For instance, the community did not show any concern if a woman had to deliver under a tree because a doctor was not available at the health facility, or if a maternal or newborn death occurred. Moreover, members of the community were unaware of their right to access public health services or their right to monitor these services and demand better quality care. As a result of prevailing traditional norms, communities tended to discount the need to invest in women’s health and well-being.  **[Increase in education and/or communication increases understanding] Mullany**: Based on these findings, it is difficult to speculate on whether the benefits seen from including husbands were a result of simply educating men (as primary decision makers in families) or educating men with their wives. Increased communication and interaction between couples regarding health practices during or after the education sessions may therefore, have led to a greater understanding and/or retention of new information  **[Role of female literacy] Turan:** Female literacy courses appear to have greatly contributed to women’s education in both areas during the time period, with around 41% of all participants having attended such a course at final assessment, compared with 27% at baseline  **[Lack of awareness of the problems and responsibilities ]Ahluwalia**: Stakeholder participation in decision making and program actions was part of the program. In the beginning of the intervention, it appaered that although maternal mortality was high, maternal health was not a priority for the communities (stated in method section, not further specified, it seems that they mean it was seen as a responsibility for families). After the intervention, community perceptions on ownership on certain barriers had changed: e.g. the arrangement of transport for obstetric emergencies was regarded a community responsibility and not of a family. After the intervention, women attended community meetings on health related topics more often and sometimes participated in decisionmaking. No report is made on how this participation or change because of the intervention was perceived.  [**Believes hindering use of services] Awhualia/Kaharuz:** Prevalent beliefs and values limit the use of delivery services. Though complications of delivery were adequately identified, understanding of their causes and management of emergencies were not appropriate and this affected the community management of these complications. For example, “Luwikilo” is believed to be a result of having intercourse with another man besides one’s husband. Complications like obstructed labour, retained placenta, and eclampsia are poorly understood and related to “Luwikilo”. [FGD women-Luhala] Mothers do not announce onset of labour - they believe that if they talk, the labour pains will disappear. [FGD men- Mbarika] A pregnant woman should not cross a river as she may abort – this may be a factor that affects use of services in an area with rivers during dry season. [FGD Men- Mwadubi |
|  |  |  | [**Mens knowledge lacking] Ahuwalia/Kaharuza**: other constraint is the delay in decision making at the community level. Focus group data showed that men are the main decision-makers who may cause delay in seeking transport given that they are either not at home or are not aware of the emergency. Some factors that limit male involvement identified included culturfs, sayings and taboos, absence from home, feeling of shame if seen supporting his wife, and lack of money. Eclampsia is also believed to be due to multiple partners and is treated by taking sand mixed with water in a glass. The sand should be from place where a dog has delivered [Men Mwabachuma]  [**Limited** **perceptions of need SBA] McPherson**: Limited knowledge of who provides local skilled birth attendance services and a preference for home-deliveries likely contributed to the low use of SBAs, as “no service available nearby” and “no practice in the com- munity” were frequently cited. Key-informants in Siraha reported that many com- munity members believe that SBAs are only necessary if an emergency occurs during delivery and, thus, only contact them in the event of a crisis. This study did not specifically explore the rationale underlying community preference for home-deliveries; however, it does not appear to be rooted in negative attitudes towards the use of health facilities. Both mothers-in-law and pregnant women reported that fathers-in-law were influential in taking decisions for issues relating to finance and transport—both related to the use of SBAs. The low use of SBAs in Siraha results from factors that include the following: faith in a traditional system of delivery care that views delivery as a natural event that takes place at home; a view of modern health services and SBAs as a ‘last resort’ to be used only if an emer- gency develops; a cadre of poorly-trained SBAs who do not possess the requisite skills and who are generally unwilling and/or unable to attend births at home; a populace with inadequate information regarding who the SBAs in their areas are and how to access them; and high costs associated with emergency transport and health services.  **[Maternal health behaviors rooted in cultural traditions] Kumar** Within communities, there is a preference toward care-seeking from unqualified medical practitioners as the first point of care, including care for obstetric complications. Maternal health behaviors have been largely shaped by deep-rooted cultural traditions and are sustained by closely knit, often caste-based social structures, the poor social status of women, low literacy rates, and limited interaction with the formal healthcare system.  **[Educational level]: FCI Tan** Consistent with the baseline survey, educated women are more likely to deliver in a facility than uneducated women.  **[Normally services not provided/task shift] FCI Kenya** It may be that more time is needed to build the communities’ confidence in mid- and lower-level health facilities given that delivery care has not traditionally been provided at many of these sites.  **[In gods hands] FCI KEN/Moore** Many community members perceive pregnancy outcomes as predetermined by God. Others commented that the time of day that labour begins determines their decisions about place of delivery  “with pregnancy, God alone will take care.” |
|  |  |  | **[Value of TBA:] FCI Ken/Moore**In other words, skilled caregivers were perceived to be better equipped and trained to provide specialized treatment for problems perceived as medical in nature, whereas community-based providers were perceived as having unique expertise in managing problems that fall outside the realm of western medicine.  **[Discussing brings misfortune] FCI Ken/Moore:** While most respondents reported that they talked about pregnancy and childbirth with family members, a few reported that these matters are not discussed. Of these study participants, a small number reported that taboos or traditional beliefs restricted such discussions, noting that discussing and announcing pregnancy could invite misfortune, putting the health and life of the woman and her baby at risk.  [**Misfortune to prepare] FCI KEN/Moore:** “You cannot prepare for something you have not seen,” argued one woman in Homabay. Another woman in Migori commented: “It is only after you have delivered is when you can buy baby layette. But when I have not delivered, there is nothing that I buy.” Some older respondents also believed that it was inadvisable to purchase items for the baby before it is born. They commented that if towels, napkins and other baby things are purchased in advance, the family will not know what to do with the items if the baby dies at birth. In addition, some saw such preparations as inviting misfortune. staff at local health facilities indicated that their counselling on birth preparedness is primarily focused on preparing items for the baby  Extra articles  **[Evil spirits in evening limiting mobilization] Choudory Bangladesh:** It is generally believed among the extreme poor house-holds that evil spirits are more active in the evening, at noon and at night, so pregnant women avoided leaving their houses during those times. Most of the respondents men- tioned that lunar and solar eclipses could affect pregnant women. They reported (those who got eclipse during last pregnancy) that they had stayed inside the household, walked near the home or inside the home, but had never laid down on the bed during eclipses.  **[Preparing birth attendant can cause black magic] Choudory** Most women did not contact the birth attendant who is locally known as traditional birth attendants (TBAs or dais) in advance because they thought TBAs could make some jadutona (Black magic) in advance during pregnancy so that without their presence delivery would not occur and that there were greater chances to pay more for delivery.  **[Preparing not necessary] Choudory** The women believed that it was bad to buy new clothes or make too many plans in advance for the new arrival as it could bring bad luck. Moreover, they were not sure whether the coming child would survive or not. Money spent on her/him was considered to be unnecessary. Women assumed that transportation would be available either from a family member or from a neighbour when needed and, as such, did not plan for the transportation in advance. |
|  |  |  | **[Importance of ANC] Magoma** Antenatal care is highly regarded in both communities, the quality of available care is considered good and most women feel obligated to attend. Regular ANC attendance is believed to guarantee healthier pregnancies and uneventful deliveries, and women who miss visits are con- sidered at risk of poor pregnancy outcomes  **[Delay in facility care for birth] Magoma** Delivery care at health units is usually sought as a last resort after serious complications have developed. Barriers identified as detracting from women’s ability to access skilled delivery and emergency obstetric care include: 1) distance from health units 2) lack of reliable and affordable transport 3) lack of advanced planning for accessing delivery care units 4) widely held beliefs that pregnancies labeled as ‘normal’ during ANC visits will result in successful deliveries at home 5) failure of providers to convey information about the importance of skilled delivery care for all women, and 6) women’s low social status and inability to independently make labour and delivery decisions.  **[EDD knowledge] Magoma** One specific communication problem mentioned was providers’ failure to inform women about the meaning of the expected date of delivery. Women in the two communities are interpreting the date listed on their antenatal cards as their actual delivery date and are waiting until this time to make delivery plans. As a result, women who start labour before their expected delivery date often end up delivering at home even if they expressed interest in delivering in health care facilities.  **[Husbands decision making] Magoma** Husbands typically decide on place of delivery in both ethnic communities, although the expressed preferences of Watemi women are beginning to be respected. Most Maasai women will only leave their households during labour after being granted permission by their husbands.  **[Naturalness of home births] Magoma** Perceptions about the ‘naturalness’ and safety of home delivery is an obstacle to convincing women in the two ethnic groups of the importance of skilled delivery care in all cases. Although the women, TBAs and Elders from both communities expressed awareness of the potential risks of delivering at home, they stressed that delivering at health units is beneficial only for women with known complications. Women with “normal” preg- nancies – defined by the women participants as those with no problems or risk factors identified at ANC visits - are expected to be able to deliver without incident at home.  **[Gods choice and complication as punishment] Magoma** They claimed that only ‘God’ can pro- tect women from a maternal death. These sentiments are rooted in traditional beliefs in both communities about pregnancy complications and maternal death as punishment for past transgressions:  **[No perceived need of SBA] Taleb** With few exceptions, women preferred birth with a TBA at home and did not recognize the value of the presence of a skilled attendant. Rarely were actions taken by women and families to prepare to access skilled care. TBAs considered themselves the principal provider of MNH services, as did women and families. Men generally felt that pregnancy and birth were a woman’s affair only and therefore remained hesitant to be involved, while still being the primary decision-maker, as the gate-keeper of family finances and women’s movement outside the household. All of these factors contributed to a low level of seeking and utilization of health services, and the program aims to influence these underlying factors to improve the health of women and newborns. |
|  | Attitudes regarding programme acceptability, appropriateness and credibility | Women and their families may have opinions about the healthcare issue and the intervention, including views about the acceptability and appropriateness of the intervention and the credibility of the provider and the healthcare system. E.g. People may not agree with the choice of intervention or may not trust the reasons behind it | **[Attitude regarding male involvement and its importance] Sinah**: Husbands, for example, perceived that pregnancy-related messages were relevant only for their wives or other women, and many refused to participate in discussions during home visits. Most husbands were willing to allow their wives to obtain antenatal care services, many would readily provide for the purchase of medicines and some even accompanied their wives to the doctor, especially those who went to a private doctor. However, the large majority perceived that their involvement and responsibility ended there; they were unwilling to assume a more proactive or supportive role at home and appeared embarrassed to discuss pregnancy-related issues with community organisers, directing them, rather, to their wives or mothers. In order to break down this reticence among husbands and involve them in discussions, the project initiated a strategy other than home visits to reach this group. Meetings were held outside the home that brought together husbands and gave them space to open up and discuss maternal health issues, including ways in which they could support their pregnant wives.  **[Mistrust in the intervention approach] Hossain/Barbey:** The failure of this CmSS seemed to be due to: 1) lack of ownership in the CmSS by a majority of families in the village CmSS; 2) lack of use of the CmSS for EmOC by the villagers; 3) lack of trust by the village as the committee running the CmSS never met; and 4) due to a lack of a transparency regarding the fund management. A fourth possible factor is familiarity with credit schemes. Many of the communities had other clubs and committees, such as youth clubs and Grameen Bank fund, and so had a good understanding of credit schemes and group process. However, as discussed above, many villages had negative experiences that were detrimental to establishing support systems.  **[Visual aids easy to understand] Hossain/Barbey**: The cards were found to be easy for the villagers to understand. In two villages the team showed them to women who had never seen them and they readily picked out salient points either on their own or with minimal orienting questioning. Two visual cues were not understood: the crosses through things a women should not do had to be explained (and the pills were mistaken for vitamins), and the visual display of time. However, with explanation, the cues appeared to be easy for women and their families to comprehend and remember. Also, the VAW flashcards have been found to be very effective.  **[Acceptability of the impementers] Mushi:** “When SMPs visit us at home we feel good because we have enough time to discuss and to ask questions“ (a nursing mother). Acceptance of volunteers by the community is important for program success. In general, the majority of respondents, including health providers, did accept and were positive about SMPs and their role in the community. In this study the inclusion of men (almost 50%) and religious leaders in the SMPs’ team made the intervention valued and acceptable.  **[Visual aids usefull] Mushi:** Nearly eighty percent of respondents visited were satisfied with the education and information, education and communication (IEC) material given  **[Visual aids need to be consistent] Sood Nepal:** It is important that IEC material is consistent so that messages are clear. Majority of respondents understood the messages which were spread.  **[Community approach to finances not matching reality] Sood Nepal:** Financial decision making and financial arrangements are more a family issue than a community issue and thus not necessarily benefitting from community level participation. |
|  |  |  | **[Acceptability of intervention across audiences] Sood Indonesia:** As a concept the SIAGA campaign had great appeal because of its applicability across a variety of audiences, with appropriately tailored goals and approaches. Husbands were specifically targeted (Suami Siaga). Level of recall of messages was remarkable, 3/4^th^ of the respondents had used the messages, the respondents were able to apply the SIAGA information to their lives, and additionally it sparked attention within the respondent’s socials networks.  [**Acceptability of the visual aids and intervention approach] Skinner:** All the village volunteers and midwives inter- viewed were very positive about the process, and many stated that they would continue to use the posters for education after the project ended. They felt that the information on the danger signs of pregnancy was new to most village members and that most understood them. The focus groups were also very successful and were one of the most positive aspects of the intervention. All were fully attended and, according to the village volunteers and the supervisory staff interviewed, were easy to organize. There seemed to be considerable interest in the community for discussing the health of their pregnant women. The midwives had never con- ducted focus groups before and although they were very nervous of doing them, all the midwives interviewed commented that the focus groups were the best part of the project as it was the first time they had been able to talk with the community.  [**Perceptions on problems] Ahluwalia** Key findings from a baseline evaluation what were causes of delay in emergency obstetric care were used to develop pogram content. (perceptions of communities on the problem used). The key findings were: lack of options and money for transportation to health facilities delayed the receipt of timely services, obtaining transport was considered a family rather than community respon- sibility, hiring transport was very expensive, and very few pregnant women were referred to health facilities. In addition, the initial prenatal visit to any health care facility often occurred after the 5th month of pregnancy when a pregnancy begins to show. On the other hand, most communities had a history of organizing and mobilizing resources to address health related problems (e.g. building a water tank for a local health facility)/  **[Accepance of implementers by community]** Awhualia/Kaharuza: VHWs in some communities enjoyed the support of local leaders and were given audience at village meetings and gave birth planning, family planning, and STI education talks |
|  |  |  | **[Male involvement poor] Ahwalia/Kaharuza:** Male involvement in reproductive health is low. CBHRP targeted pregnant mothers and expected husbands to attend birth-planning discussions. VHWs reported this rarely occurred.  **[Understanding BPCR messages] Moran:** women understood the components of birth-preparedness and complication readiness and mentioned antenatal care services as a means for ensuring good health for their babies. They heard of the concept from auxiliary mid- wives during antenatal care visits and from radio trans- missions.  [**No perceived need to prepare transport] Moran**:Although many women planned to give birth in a health centre, few women made plans for transportation. Women assumed that transportation would be available either from a family member or from a neigh- bour when needed and, therefore, did not feel the ne- cessity of planning for transportation in advance. A 35-year old explained: “I do not have any mode of transport. But there are certain people in the family who will help bring you in case you need. I thought that, at the time, I would feel the labour starting, within the family, I could find someone who would take me to the health centre. Since we live together, there are those who have a method of transportation and she goes with them.”  **[Acceptability of approach] McPherson**: Key-informant mothers-in-law and pregnant women reported that most ‘women like us’ understood the messages and found them to be acceptable. These informants confirmed the survey results by reporting that key chains—FCHVs, trained TBAs, and other health workers—were important channels for receiv- ing the BPP messages. They noted that some pregnant women prefer trained TBAs to FCHVs as Mobilizers because they also deliver services.  **[Neonatal issues more important] Hodgin** perception that effective care for neonatal problems may be more affordable and more readily at hand than care that would be required for maternal complications.  **[TBAs forming culture bridge] Fonseca:** At Sololá Hospital, TBAs are allowed to accompany women during childbirth, providing social support in a culturally sensitive manner. Anecdotal data suggest that nearly all women giving birth at Sololá opt in for this practice, as they often do not speak the same language as the formal medical providers and it is not part of their usual practice to have their husbands join them in the delivery room.  **Extra articles**  **[Roles of HCW overlap] Choudry** it was found from the present study that since the same individual was responsible for providing contraceptive pills as well as for antenatal care, the women felt shy and sometimes scared to share their pregnancy news with the health care provider in fear of being scolded for discontinuation of the contraception  **[Low expectation of care] Magoma 2013** Women have low expectations of care so satisfaction can be a difficult measurement |
|  |  |  | **[Acceptance of information] McPherson** Mothers, mothers-in-law and almost all husbands who were interviewed perceive information given by their FCHVs to be accurate and trustworthy. Several respondents said they would have preferred to receive more detailed information on where to go for delivery and how to arrange for transportation. Some mothers mentioned that they would prefer if the FCHV would promote messages from the booklet with their family members more aggressively as it is difficult for mothers to influence senior family members regarding care-related decisions. For example, one mother said she would have preferred that the FCHV convince her mother-in-law to arrange for a skilled birth attendant  [**Acceptance for both literate and illiterate] Taleb** Participants expressed that this change has been facilitated by utilization of the BEPP card which had been introduced in the study area by CHWs of partner NGOs. All participating women were aware of the BEPP card and had used one to elab- orate a plan for birth and complications. Women had received their cards from CHWs participating in the program or from health care providers when having sought ANC in health facilities. The card was deemed to be effective in transmitting information to participants regardless of literacy status and illiterate men and women expressed an in-depth understanding of the elements illustrated in the card.  **[Fear of having blood withdrawn] Taleb** as a reason for non preparation for blood donor. |
|  | Motivation to change or adopt new behaviour | Women and their families may have varying degrees of motivation to change behaviour or adopt new behaviours, e..g. they may be more or less motivated to seek care | **[Lack of husbands motivation to change] Sinah**: Changing husbands’ attitudes was not an easy task. It was clear that efforts to make husbands more supportive questioned deep-rooted norms and beliefs, and met with considerable resistance; consequently, husbands were slow to change their views. Attendance at group meetings appeared to enhance solidarity among husbands, many of whom agreed to counter traditional norms and contribute to housework; reduce alcohol use and spend, rather, on improving the nutritional status of their pregnant wives; and accompany their wives for pregnancy-related services. Often, during meetings, men who had attended one or more previous meetings described the ways in which they had supported their pregnant wives, providing role models for other husbands as well. However, several women reported that while their husbands were willing to contribute to housework, they were apprehensive of the reaction of their parents, neighbours and others to men becoming engaged in what is perceived as women’s work. There is also a need to address deep-rooted traditional gender norms that underscore that pregnancy is a “woman’s issue,” and that husbands are not required to participate in pregnancy care.  **[More motivated to change if concrete role for husband, also more motivation if mother in law is not there] Mullany**: Men’s roles during the post-partum period are traditionally more limited than in the antenatal period because female family members more commonly assist the woman at this time [51]. It is possible, therefore, that taking his wife to a post-partum checkup may provide a concrete way for a husband to feel less ‘left out’ and to become involved during the post-partum period. Alternatively, husbands may perceive the post-partum checkup as being important for the baby, and may therefore express greater interest in this after learning about its importance. Since the time of birth has traditionally been viewed as mothers- in-law’ domain in much of South Asia [45], it is conceivable that women (and their husbands) living apart from a mother-in-law are more receptive to learning about birth preparedness messages because they know no one else is there to take care of such matters.  **[Hiding of pregnancy reduces motivation to change but intervention seemed effective to tackle this] Mushi:** Due to culture and beliefs, women in many settings do not disclose their pregnancy status and most of them wait until the third trimester. In our study area, early visits by SMPs made it possible for more women to disclose their pregnant status and to book early for ANC. Although not all women were will- ing to disclose their pregnancy status to SMPs during home visits, as soon as the SMPs had left, a number of women with early pregnancies decided to go to ANC, based on the SMPs general advice and considering that in the following months the “hidden secret” will be known anyhow.  **[Women feel the danger of home birth] Moran:** Most women planned to give birth in a health cen- tre with a skilled provider to ensure ‘health security’. Women stated that complications can arise at home, and health workers in the health centres are more quali- fied and competent to handle and refer these complica- tions.  **[Exposure to maternal deaths movitated communities to address the problem] Hossain/Barbey**: A third facilitating factor was the large number of maternal deaths. While not all villages had experienced a maternal death, all of the communities interviewed had heard of a maternal death in a neighboring village. However, families were only able to recognize and address the problem when CARE staff brought a case to their attention and encouraged them to identify their own solutions. These cases stimulated thinking and problems solving, motivating community members to do birth planning and preparation. |
|  |  |  | **[Carefull with insentives] Awhualia/Kaharuza**: The project provided bicycles to VHWs to allow them to cover the extensive distances implied by their catchment areas. Management of this resource proved problematic in some instances. Some communities saw the bicycles as incentives to the VHW and therefore felt no need to develop other mechanisms to compensate or “encourage” their VHW. This led to dissatisfaction on the part of some VHWs.  [**Motivated to prepare] Moran:** Women consistently mentioned saving money during their pregnancy for antenatal care, unforeseen costs due to birth-related complications, buying essentials, such as foods, soap, and clothes for the mother and newborn, and a means to avoid borrowing money from others. Women generated income for their savings plans mostly through agricultural and market activities, including selling millet, peanuts, dolo (local mil- let beer), and shea butter. Their husbands who saved money also had savings plans through agriculture and raising livestock. Several women mentioned learning about the importance of financial planning and how to save money at antenatal care visits. Healthcare workers explained a method that almost every woman revealed save half of what is earned for the day and spend the other half. A 39-year-old asserted the importance of a savings plan: “It is good, something that I thought about and planned. I say that when I knew I was pregnant that it was necessary to save five francs every time I earned 15 francs. This initiative was to help me face the needs of the expenses that would come with the pregnancy, during the birth, and even after the birth. In saving the money, this helped me to have financial means to do what necessitates in any the case of need, that is what I did.”  [**District chosen due to previous positive experiences] FCI TAN** The district was relatively small, and it had an active and committed District Health Management Team (DHMT) which had successfully piloted the Community Health Fund, a World Bank-funded community health insurance scheme. In addition, the district had an adequate health infrastructure at the outset of the project, which was an essential foundation for planned interventions  **[Fear for male provider] Taleb** For instance, women are hesitant to seek services at formal health facilities since they fear that a male pro- vider may ultimately attend the birth. According to the participants, this is culturally unacceptable. In addition, although all participants wished to give birth in the pres- ence of a CSBA at home, availability of CommunitySBAs was an issue. In situations where a CSBA was not available, the default action was to give birth with a TBA at home rather than going to a health facility, although most participants were aware that they had access to transpor- tation schemes that had been developed within the con- text of the program.  **[Decision making power] Taleb** On a promising note related to rights, women and men expressed their perception that interventions related to birth preparedness and complication readiness are influencing the role of women in household decision-making processes which was previously limited to male members. Interactions between women and their family members in decision-making were reported to be present in terms of seeking health care from informal or formal providers. Though women’s participation in decision-making processes is reportedly increasing, informants also stated that women are still not the primary decision makers as the final decisions are made by the male members of the women’s family.  [**Intention leads to action but not in unpredictable situations] Tura** For policy and program implications, this study came up with the evidence that intention implies action in skilled care use; but sometimes this may not be the case as complication during labor is unpredictable |
| 2. Healthcare providers and volunteers involved in implementing the intervention | Knowledge and skills | Providers may have varying degrees of knowledge about the healthcare issue or the intervention, or may not have the skills to apply this knowledge. E.g. health workers may be unaware of service models to address cultural barriers to care, or may not have received training on how to implement these models | **[Community organizers had skills to bring people together] Sinah**: Most community organisers had 10 years of schooling. They were skilled in bringing people together and working with them to initiate change. They had the advantage of knowing the local residents and of having established a rapport with members of local bodies such as the gram panchayats, youth groups and mahila mandals. They had also established a reputation for being honest and dependable, which contributed to the acceptability of the intervention activities. Prior to initiating the intervention, community organisers underwent training on issues related to maternal health. Through these organisers, the project established a link between pregnant women, their families and the community; pregnant women and the health system; and the community and the health system. Community organisers supported youth committee leaders to build awareness among youth of the need to make health service providers accountable to the community.  **[Less knowledge than expected] Bagui:** Through interviews with programme managers and community- based workers, we identified that the workers’ competency in the new neonatal component of the programme, their workload and inadequate management and supervision were possible barriers to higher coverage. CARE-India conducted an evaluation and noted that change agents’ reach was lower than expected.  **[CHW need adequate training]** **Darmstadt**: Intervention was designed to address the major causes of mortality in neonates, it was most robust for the prevention and management of infections. In the Mirzapur population, however, nearly 60% of deaths were due to birth asphyxia or prematurity, and the program had limitations in reaching households at the critical times (i.e., during labour, childbirth and immediately after delivery) to address these conditions, and the CHWs lacked the necessary tools and skills to effectively address these conditions  [**Selection of implementers] Kumar** The primary enablers of behaviour change were paid (US$35–40 per month) community-based health workers, the Saksham Sahayak (n=26), who were recruited from the local community based on 12 years or more of education, proficient communication and reasoning skills, commitment towards community work, and references of community stakeholders.25 |
|  | Attitudes regarding programme acceptability, appropriateness and credibility | Providers may have opinions about the healthcare issue and the intervention, including views about the acceptability and appropriateness of the intervention and the credibility of the provider and the healthcare system. E.g. health workers may not agree with the choice of intervention or may not trust the reasons behind it | **[Job satisfaction] Ahluwalia**: 27% of VHWs reported low satisfaction; 62%, medium; and 2% high.: The fact that VHWs have begun to organize themselves indicates their com- mitment to their work and the value placed on it by their communities.  **[No acceptance of full tasks] McPherson** Although the FCHVs were encouraged to promote the BPP messages through home-visits, these visits are not part of the job description of the FCHV, and some FCHVs were reluctant to perform this task.  [**BPP messages not concrete enough]McPherson** The BPP personnel reported that the BPP was helpful in arranging existing messages into a coherent framework. They noted that some new messages were difficult to pro- mote because they lacked concrete, pragmatic mechanisms to achieve their performance. The broad focus of birth-preparedness messages should be matched by an equally broad array of activities to support their practice.  [**Appreciation for work] Skinner**: Moreover, for the staff of the MCH service in Cambodia, it was a satisfying and exciting project, in part because it established their own capacity to undertake such work, unassisted by NGOs. The findings are a reflection of the fact that making meaningful change need not be expensive. The enthusiasm at all levels was apparent, from national MCH leaders to village volunteers.  Extra articles  **[Time of ANC] Magoma** The providers interviewed cited heavy workloads as the main reason for insufficient dialogue on the importance of skilled delivery care.  **[Feeling appreciated] McPherson:** FCHVs in Nepal are generally recognized and honored by the communities they serve for their contributions. Elderly FCHVs who served as respondents noted that they do not want to retire from their positions due to the appreciation they receive from the community. Some FCHVs stated that now they are trusted more than before the intervention by their clients due to the new services they provide. Other FCHVs expressed the pride they feel in providing women and children with information and services. One FCHV noted that the enhanced supervision that she received under the intervention motivated her to work harder.  **[Cards good but too many messages] McPherson** Too many messages and repetition of messages causes some cards to be skipped, reducing the amount of messages might make it better used.  **[Fear of costs discussion] Soubeiga 2013** However, the proportion of women in this study who received cost information was low. It appears that care providers were reluctant to talk about cost of care with prenatal patients because the rates set by the Ministry of Health are not applic- able to all patients. |
|  | Motivation to change or adopt new behaviour | Providers may have varying degrees of motivation to change behaviour or adopt new behaviours. E.g. they may be more or less motivated to take on new tasks | **[Motivation of men to take on change agent role] Mushi**: In our study we have been able to demonstrate that men can be organized as a group of change agents that work hand in hand with other community structures in promoting safe motherhood. Half of the SMPs team was men. Since, in traditional societies, men are respected, heard and they make or influence most decisions, the involvement of men in the SMPs team was an important element that contributed to the effectiveness of the intervention. Involving men in maternity care is essential and health planners should be trained to view men as “partners and key players” and not as “barriers”. A positive view of men will increase cha  Extra articles:  **[Time constrains] McPherson** FCHVs reported difficulties finding the time to complete their newly assigned tasks given their per- sonal responsibilities; FCHVs who serve scattered popu- lations or who cross rivers on foot during monsoon to reach clients were especially vocal in this regard. |
| 3. Other stakeholders (including other healthcare providers, community health committees, community leaders, programme managers, donors, policy makers and opinion leaders) | Knowledge and skills | Other stakeholders may have varying degrees of knowledge about the healthcare issue or the intervention, or may not have the skills to apply this knowledge. E.g. a community leader may have insufficient knowledge of the benefits of community participation in quality improvement or may not feel skilled in running community meetings to promote this | **[Increase in knowledge and skills of other stakeholders due to intervention leading to cascade of influencing relevant community stakeholders] Sinah**: Gram panchayat and youth committee members reported that, following project activities and review meetings, they had become sensitised to consider health an important community need. Gram panchayat members and youth who could influence families and women as well as demand accountability from health service providers, ANMs who are directly involved in providing health services, and teachers and headmasters who are respected members who could mobilise attitudinal change.  **[Increased understanding between community, facility and leadership of the problems women experience] Sinah**: Community leaders reported that as a result of direct interaction with government officials on problems faced by health providers and the women themselves, there was more openness among officials to resolving issues and a greater willingness to accept feedback from the community. For example, at review meetings, one problem repeatedly highlighted was that several women were being unnecessarily referred to the District Hospital when they sought deliveries at the primary health centre. Gram panchayat members brought this problem to the attention of the primary health centre staff and were apprised of the poor facilities in the primary health centre and the inability of the staff to cater to unforeseen delivery-related complications; at the same time, the medical officer assured them that every effort would be made to minimise unnecessary referrals. Such interaction not only led to a better understanding between community members and primary health centre staff, but also motivated communities to apply pressure on government staff to provide the necessary services at the level of the primary health centr . |
|  | Attitudes regarding programme acceptability, appropriateness and credibility | Other stakeholders’ may have opinions about the healthcare issue or the intervention, including views about the acceptability and appropriateness of the intervention and the credibility of the provider and the healthcare system. E.g. stakeholders may not agree with the choice of intervention because of competing interests or priorities | **[Other HCW accepted the promotors of the intervention] Mushi:** SMPs were also accepted by health providers and they felt proud when realizing that their work was valued and supported by professional health workers. Some- times SMPs would meet with midwives to ask questions and to discuss progress and challenges. This was possi- ble because from the beginning midwives were involved in all stages and in the main activities of the intervention. Midwives also supported SMPs because they found them to be helpful in their community outreach activities. Health providers and community leaders were also found to have a positive attitude towards SMPs. SMPs reported that they were very much accepted by the midwives in the health facilities and that they were free to meet them in case of any question.  **[Positive attitudes of drivers and of men to increase assistance to pregnant women] Sinah:** Auto rickshaw drivers were also mobilised to provide transport without delay, and on credit if necessary, to pregnant women. As all members of village level youth committees were men, community organisers helped youth groups to set up committees of girls and young women. Interestingly, members of male-dominated youth committees helped to overcome the obstacles to girls’ participation at these meetings.  **[Leadership view of intervention promotors] Ahuwalia**: All village leaders and villagers appeared to have a good understanding of the VHW’s roles and responsibilities. Three villages indicated that VHWs were not providing reports on their activi- ties, 38 that the VHWs reported to the village leadership on occasion, and 11 VHW’s reported regularly. More than half (32y52, 63.5%) reported that VHW reports were used to help guide decisions.  **[HCW accept implementers] Ahuwalia/Kaharuza**: Health facility staff recognized and acknowledged the roles of VHWs and TBAs in referral of mothers to the heath units. Health facility staff also trained TBAs in safe delivery at the health facilities. Health centre staff carried out support supervision visits to TBAs  Exra article  **[Positive perception of jobaids] Jennings** The three most commonly reported advantages to using the aids were that they helped women retain information given the images of key signs or practices; they helped the provider remember what topics to discuss during the antenatal session; and the perceived time required for explaining a practice was less since the images well depicted the desired communication goal. sing the counseling job aids also noted that having them allowed workers to improve their skills over time and that women presenting at the clinics also appre- ciated and showed interest in the counseling cards. On the other hand, providers remarked that the required additional time to use all of the counseling cards in a given module, including verification of prior knowledge in preceding modules, was a disadvantage that delayed women’s departure from the antenatal clinic. Some providers suggested that the number of counseling cards be decreased in tandem with an increase in the number of messages per card while improving their durability as well from the original laminated format. A few workers proposed that the training session be extended from its current three-day module to provide more time for discussion and prac- tice. All providers recommended that the counseling job aids be introduced at other sites to strengthen antenatal education. |
|  |  |  | [Continuation] During interviews, providers reported that lack of or limited dedicated space for counseling remained a challenge along with language barriers (in areas with multiple dialects). In addition, clinical tasks, such as managing deliveries, disrupted or prevented high quality antenatal communication. Lack of time was the most commonly reported barrier as providers recog- nized that good communication takes time. Particularly in facilities where health personnel are understaffed or have multiple clinical responsibilities, one alternative may be to explore the feasibility and effectiveness of expanding the role of less skilled health workers who may have fewer time constraints. Nearly three-fourths of women in the intervention arm said they would have preferred having the provider spend more time with them for counseling. One drawback may be overreliance on the tools that results in overly structured, less individualized sessions.  [**Staff difficult in ending project] Skinner** The time-limited nature of the project caused considerable distress to the planners and imple- menters when they realised that the work they had done was going to cease as the project ended. They were particularly concerned at the loss of the midwifery workforce, either to unemployment or back into the private sector. It proved challenging for the Kampong Chhnang staff to get ongoing funding for further production of the posters or to continue with community engagement. What did eventuate however was a series of requests from other districts, some of which were managed by NGOs, for use of the posters and the training packages. |
|  | Motivation to change or adopt new behaviour | Other stakeholders may have varying degrees of motivation to change behaviour or adopt new behaviours. E.g. programme managers may not be motivated to supervise TBAs in new roles | **[Motivation of TBAs to change practices]** **Mushi:** The intervention has built linkages between health providers, SMPs and pregnant women and TBAs. TBAs who were part of SMPs have become active promoters of skilled attendant at delivery and some have changed from delivery care provider to educators and counselors and referral advisors. Further- more being part of the team some TBAs’ became reluctance to perform home deliveries on their own, particularly in women with specific risk factors  [**Being open for others] Hossain:** stepwise implementation of stakeholder committees sensitized providers to the importance of community involvement and enabled community members to be active participants in decision-making  Extra articles  **[ANC time] Jennings** These improvements were associated with increases in consultation duration. Each session lasted an average 18 minutes at baseline in both study arms and significantly increased to 24 minutes in the intervention arm (ΔI - Δc = +5.9, 95% CI: 3.0, 8.8). The observed additional time appears to have been associated with increased communi- cation, although time spent in clinical examination versus communication was not measured systematically. |
| 4. Health service delivery factors | Accessibility of care | The accessibility of healthcare facilities may affect implementation of the option, for instance because of financial (user fees), geographic (distance to clinic), or social (access for certain ethnic groups) factors | **[Lack of perceived access] Skinner**: there were also negative comments relating to the affordability, accessibility and acceptability of the health centre and its staff. These related to the fact that health centre care was more expensive than TBA care, the centres were not open all day or at night, and fear of the staff, who were often unknown. The voices from the community revealed that although there was movement towards understanding the causes of maternal mortality, there was still a real need to address issues of accessibility, affordability and acceptability of both midwifery care (midwives should be known and trusted) and health centre care birth (it needed to cost less and be open 24 hours a day, seven days a week). In the participating health centres, there was a tendency for staff to only work in the public sector in the mornings, with the afternoons being for their ‘private’ cases. This left the health centres unattended by midwives and seemed to be a well- accepted practice.  **[Elliminated barriers to access] Darmstadt**: To eliminate potential barriers to care seeking for illness, CHWs facilitated transport, if necessary, for neonates needing referral-level evaluation at Kumudini Hospital, and all care at the hospital was free-of-charge for referred neonates. The mean travel time to the hospital was about one hour,and formative research suggested positive community perception of the quality of care at the hospital.  **[Large geographical distances making implementation difficult] Ahuwalia/Kaharuza**The project staff noted that, since villages are so large (often consisting of five or more sub-villages) it was very difficult for just two VHWs to cover their assigned area. Health facilities are geographically inaccessible. The roads are poor, especially in the rainy season and villages are remote from even very basic care. Again, remedies for this problem were beyond the scope of the project. Referral to the tertiary facilities remains a challenge. VHWs refer to the nearest health facilities, but this level often finds it difficult to refer to next level, due to lack of communication and transport.  [**Large distances] Moran**: Distances from villages to health centres were often long; women travelled 22.8 km, on average, through remote, rural areas to reach a health facility. One woman stated: “I gave birth at home because the health centre is far away and it was during the rainy season. During the rainy season, two bodies of water enclose us and since it was during the night, if I went to the health centre, I would not be able to arrive there... even if you are with a moped, during the rainy season you cannot arrive. The person risks giving birth on the way and, there, that would be me on the way.”  **[No accessible care] McPherson** ANMs and MCHWs are seldom asked to attend births. Services provided by these workers are supposed to be free, although informal charges of US$ 10-20 may be levied. There is one government hospital in Siraha municipality that offers 24-hour delivery services but does not provide basic emergency obstetric services. Women in Siraha who require a caesarean section or management of severe complications have a choice of using one of two facilities offering CEOC services in Lahan, Siraha—the government hospital or a private nursing home—or travelling to a neighbouring district/Most women in Siraha must travel 2-8 hours by oxcart and/or taxi to access these services at a transportation cost alone of US$ 5-30. Borghi et al. noted that the average cost of a caesarean section in a representative cross-section of districts in Nepal—in- cluding service, transport, and opportunity costs, and additional charges—exceeds US$ 150. Thus, while the SBAs—as categorized by the Government—are relatively accessible, facilities and personnel that offer basic lifesaving services to a woman with an obstetric emergency are much less so. |
|  |  |  | [Continuation]The BPP messages must be consistent with avail- able health services. The lack of supply-side program- ming in the Siraha BPP field trial represents a central limitation of the Programme design. Care-seeking deci- sions take place within a complex setting that includes community-level factors, such as characteristics of local health systems. Birth-preparedness programmes will be more effective if they improve accessibility to and quality of health services.  **[Improving accessibility, quality] Fonseca**: Working in close collaboration with the MOH, the Program developed a Performance and Quality Improvement (PQI) approach using an accreditation model. The approach ensured that there were facilities (hospitals, community maternities, health centers, and health posts) that had skilled personnel, as well as supplies and management systems, in place so that obstetric emergencies could be attended to. It should be noted that not only was the clinical aspect taken into account, but factors such as interpersonal communication, infection prevention, and issues such as who could accompany the woman during birth were also considered.  **[Capacity of HF perceived low] FCI KEN/MOORE** Study participants also commented on staffing shortages at local facilities and observed that it can be difficult to locate a skilled attendant at the health facility when one needs information and treatment. They added that there are usually too few skilled attendants to provide effective services, especially when women present with complications.  [**QoC low] FCI KEN/MOORE** many characterised facility-based providers as negligent at best, and as emotionally and physically abusive at worst. Others complained of outright neglect, describing health staff as inattentive and unconcerned about women’s progress with labour or their discomfort. Community members also perceived facility-based staff as judgmental and discriminatory, commenting that women who are well- dressed receive good care, whereas those who appear less affluent are shamed and criticised.  **[Distance/transport] FCIKEN/MOORE** Inaccessibility of facility-based delivery care: Community members cited distance to health facilities and lack of transportation as major barriers to use of skilled care. Most community members described the hospital as being very far away, and they spoke of travelling long distances—usually on foot—to reach a facility. The problems of distance and transport appeared to play a major role in determining where a woman would deliver. Many study respondents indicated that while they would prefer to deliver at a health facility, they did not even consider this a realistic option.  **Normally services not provided/task shift] FCI Kenya** It may be that more time is needed to build the communities’ confidence in mid- and lower-level health facilities given that delivery care has not traditionally been provided at many of these sites. |
|  | Financial resources | Additional financial resources may be needed to implement the option | **[Other barriers limiting impact] Darmstadt**: mphasis must be placed on community mobilization and empowerment,[48] and on greater understanding of and development of improved approaches to overcome social and financial barriers to referral compliance and care seeking at facilities, especially in the first week of life and in settings where cultural seclusion after birth remains a social norm  [**Financial support for implementers] Ahuwalia:** Initially, villages did not provide any social, financial, or technical support for VHWs. After CBRHP, however, most villages provided techni- cal, administrative, and social support for VHWs, and some occasionally provided financial support. Our data show that VHWs enjoyed strong social and administrative support.  **[High out of pocket expenses limiting access] Awhualia/Kaharuza**: It is a reality of the current economic situation in Tanzania that there are high informal charges at health facilities. Although maternal and child health services are supposed to be free, informal charges are common and are a deterrent to care seeking. Furthermore, the formal charges at paying hospitals were said to be out of reach for some members of the community.  **[Out of pocket expenses] FCI Tanzania**. Although health services for pregnant women are officially provided free-of-charge in Tanzania, in reality, women do incur out-of-pocket expenses for such care. Fully 89% of women in the baseline survey and 91% in the endline survey in Igunga reported incurring out-of-pocket expenditures for delivery care. Thirty-nine (39) percent of women said that the costs of delivery care were more than they expected. The large increase in cost of care during the intervention period may have been an important barrier to skilled care-seeking, even those who were exposed to the SCI intervention.  **[Out of pocket expenses] FCI KEN.** In both surveys, the vast majority of women (97%-98%) reported that they incurred out-of-pocket costs for institutional delivery care. There was no decrease in the proportion of women who reported paying out-of-pocket expenditures for maternity care in the 2006 survey, approximately 20 months after the cost-sharing policy had officially ended. In addition, the overall costs of care increased; the mean costs of normal delivery care represents approximately 17% of mean monthly household income while the mean costs of complicated delivery care increased by 32%, from 1,791 Ksh, to 2,363 Ksh—an amount that could potentially result in household impoverishment. These high costs of care seeking may be an important barrier to the use of skilled care during childbirth.  **[Transport costs] Magoma** For most Maasai and other women living in remote villages, transport to health units for delivery or emergency obstetrical care is unreliable and unaffordable. |
|  | Human resources | An increased supply or distribution of health workers may be needed to implement the option | **[Challenging staff levels and motivation] Hossain**: In terms of quality of care, staffing during nights and weekends varied from facility to facility. Many service providers do not live nearby further pro- longing delay in treating complications. There was frequent turnover of key personnel. Finally, sustaining high staff morale and commitment amidst difficult working conditions proved difficult.  **[High workload] Bagui:** Through interviews with programme managers and community- based workers, we identified that the workers’ competency in the new neonatal component of the programme, their workload and inadequate management and supervision were possible barriers to higher coverage. CARE-India conducted an evaluation and noted that change agents’ reach was lower than expected.  **[Poor quality and staff shortage] Ahhuwalia/Kaharuza**: The quality of services was quite poor at some health facilities. Some of the health facilities were poorly equipped, lacked privacy, were severely understaffed, and staff lacked skills and/or motivation. Some of the factors were beyond the scope of the project. The project was able to train some health workers and but could not equip or improve the infrastructure of the health units. |
|  | Training | Healthcare providers, community health workers or community members may need to be trained in how to deliver or use the intervention | **[Training implementers and HCWs] Hossain/Barbey**: Training was another facilitating factor. As reported above, CARE staff facilitated hundreds of on the job training sessions which were key to educating families on danger signs and preparation for birth.  **[CHW need adequate training]** **Darmstadt** Intervention was designed to address the major causes of mortality in neonates, it was most robust for the prevention and management of infections. In the Mirzapur population, however, nearly 60% of deaths were due to birth asphyxia or prematurity, and the program had limitations in reaching households at the critical times (i.e., during labour, childbirth and immediately after delivery) to address these conditions, and the CHWs lacked the necessary tools and skills to effectively address these conditions.  **[Intervention provided training] FCI TAN** A total of 115 maternity care providers from the district were trained in routine obstetric care skills, such as focused antenatal care, active management of the third stage of labour, use of the partograph, infection prevention, and interpersonal communication skills, including compassionate care, as well as the management of obstetric complications (e.g. pregnancy-induced hypertension, haemorrhage, shock, prolonged and obstructed labour, and sepsis). A total of 76 providers (doctors, nursing officers, nurse/midwives, and clinical officers) were trained in advanced life-saving skills (ALSS), and 39 providers (Public Health Nurse Bs, trained nurses, and MCH Aides) were trained in basic life-saving skills (BLSS).  **[Lack of impact of training] FCI TAN** Evaluation results suggested that changes in provider knowledge and competencies related to the management of obstetric complications were small—results that were surprising given that all maternity care providers were trained in either advanced or basic life-saving skills, which is an intensive competency-based residential training. These results may be influenced by several factors, including the transfer and redeployment of many providers trained in LSS, as well as evaluation challenges related to measuring providers’ skills and competencies.  **FCI TAN/KEN** and Brazier all focused on increasing QoC as well with training and infrastructure improvements.  **[Job aids wih training and supervision] Jennings One**, this study demonstrates that job aids with training, field support, and organiza- tional change are an effective strategy for improving provider communication and should be integrated into routine antenatal care strategies. |
|  | Communication | Changes in communication between different levels of the health system or between the community and the health system may be needed to implement the option | **[Increasing communication between levels] Mushi**: The intervention has built linkages between health providers, SMPs and pregnant women and TBAs. TBAs who were part of SMPs have become active promoters of skilled attendant at delivery and some have changed from delivery care provider to educators and counsellors and referral advisors. Strong link between the community and health service providers through village-based structures such as the village health committee.  [**Increasing mutual understanding] Sinah:** The regular review meetings not only ensured the proper functioning of health facilities and staff, but also provided health functionaries an opportunity to share their problems and the obstacles they faced in performing their duties and providing services. This, in turn, helped the community to appreciate providers’ difficulties. |
|  | Accountability | Changes may be needed so that those with the authority to make decisions are accountable for the decisions they make | [**Increase in accountability] Sinah:** Several gram panchayat and youth members reported that the primary health centre staff were attending to their duties more regularly, and the need to be accountable to the community was better accepted by the staff. Because we did all this [questioning public health facility staff in gram panchayat meetings], ANMs and doctors come on time and do their work properly. Youth committee members likewise reported that activities such as regular committee meetings and frequent visits to the primary health centre had resulted in more accountability among health staff, better quality services at facilities and improved care by providers at the primary health centre. |
|  | Leadership roles and responsibility | Implementation may require multiple organizations at multiple levels working in partnership, and require participation and support of many stakeholders or sectors. Leadership and responsibility of each group may need to be clear | **[Partnerships with clear understanding of different roles and responsibilities] Hossain/Barbey:** One of the important values of partnership that has been practiced throughout the DSI is the feeling of ownership. CARE took the key role to facilitate and advocate for the partnership process involving all the partners, clarifying understanding of the purpose and mutual expectation from the outset of the Project. Partners included: household, community, GoB Health Infrastructure at the Upazila and below, local institutions such as local NGOs, CBOs, Union Parishad, and educational institutions, TBAs and village doctors, CARE, UNICEF, and the GoB. Each partner was involved in each step of the Project, giving a feeling of ownership of the partnership to the partner. It was believed that the less involvement of money in the partnership, the more likely the efforts will be sustained. The success of the partnership was due to the consistent practice of participatory methodologies, partnership values, and guiding principles.  **[Implementation through government infrastructure] Baqui**: The programme was imple- mented through the infrastructure of the government’s Ministry of Women and Child Development’s Integrated Child Development Services and the Ministry of Health and Family Welfare  **[Collaboration] Ahuwalia/Kaharuza**: Close collaboration among key actors in maternal health service delivery was a key determinant of project success at all levels: health facilities, the community and partner agencies.  **[Involvement of leadership] McPherson:** The Siraha District Health Office (DHO) was involved at all levels of the implementation of the Programme. The BPP-related tasks were part of the workplan of the DHO, monitoring-data were collected through the reporting system of the DHO, supervision of the BPP was integrated into the supervisory activities of DHO, and the DHO staff served as Master Trainers for the roll-out of the BPP.  [**Committed leadership] FCI TAN** The district was relatively small, and it had an active and committed District Health Management Team (DHMT) which had successfully piloted the Community Health Fund, a World Bank-funded community health insurance scheme. In addition, the district had an adequate health infrastructure at the outset of the project, which was an essential foundation for planned interventions |
|  | Information systems | Adequate information systems to assess and monitor needs, resource use, and utilisation of the intervention may be needed to implement the option | **[Increased use of data]** **Hossain:** For example, the regular use of data in the facilities, including death and near miss reviews, allowed service providers to better understand patient needs and the importance of providing timely and quality care.  **[Effective information system] Ahuwalia/Kaharuza**: The information management system developed promotes integration of data and its collection at all levels calls for extensive feedback at all levels. The feedback allows community dialogue and decision making on factors that affect maternal and child health in their communities. The participatory monitoring systems (the community board), bridges community and health facility data for public health information/data for decision making.  **[Both supply and demand side] Brazier:** The importance of both supply and demand side attention as well as focus on infrastructure problems roads etc to increase accesibility |
|  | Facilities | Adequate supply and distribution of necessary supplies and equipment to facilities, and maintenance of these facilities, may be needed to implement the option | **[Supplies shortages hindered working conditions] FCI tanzania**: Although the project included interventions to improve requisition of drugs and supplies, no improvements in these areas were observed. Routine monitoring of this issue during project implementation revealed that while facility staff were ordering the drugs and supplies they needed based on their caseloads, the items ordered were frequently not available from the Central Medical Stores Department because of logistics problems at the national level—a challenge that is not possible to address through a district-level intervention.  [**Aims to Address equipment and supply gaps] FCI Tanzania**Based on gaps identified through baseline research, a package of essential obstetric equipment was provided to each health facility — equipment such as blood pressure gauges, weighing scales, delivery kits, examination beds, autoclaves/sterilisers, examination lamps, reagents, autoclave drums, speculum, trays, baby towels, etc. FCI also worked with district health managers and facility in- charges to strengthen logistics systems and improve the availability of essential obstetric drugs and supplies. These efforts included modification of the Community Health Fund (CHF) drug ordering form to include drugs specific to maternal and child health, as well as training staff at all health centres and dispensaries in a new logistics system (the Indent System) to support the district’s shift from a kit system to a pull system in which each facility determines and orders its needs based on its caseload.  **[Skills and drugs] FCI Tanzania** The primary improvements that women reported observing at their local health facility were improvements in provider skills and drugs. Interestingly, while the availability of solar power, radio equipment or the ambulance would seem to be improvements that would be relatively apparent or visible to community members, these types of improvements were only mentioned by about 8% to 11% of women who reported noting changes at their local facility. In addition, while there was a significant improvement in the basic infrastructure (source of solar power, water source, and steriliser), there was no significant difference in the utilisation of “high infrastructure” and “low infrastructure” facilities, indicating that such improvements did not influence women’s care-seeking decisions.  [**Improve the availability and quality of maternity care through health systems interventions] FCI KEN** These interventions included upgrading the health infrastructure, including surgical facilities, where needed; addressing equipment and supply gaps; training providers in clinical and interpersonal skills in routine and emergency obstetric care; providing resources to strengthen referral systems and improve health management systems. |
|  | Bureaucracy | Paperwork and procedures may need to be structured to facilitate rather than hinder implementation of the option | NA |
|  | Intervention integrity | The extent to which it is implemented as planned helps determine why an intervention fails or succeeds. This includes quality of delivery, participant response and enthusiasm, and contamination with other concurrent interventions. | **[Government activities] Turan**: These improvements may be due to other safe motherhood campaigns of the Eritrean Ministry of Health and/or the increased educational level of women  **[Government activities and other partners activities]**: **Sood Nepal:** The government of Nepal supports many SM partners in an effort to reduce maternal and neonatal morbidity. In the area of behavioral change many partners are implementing complementary acivities. |
| 5. Social and political factors | Ideology | Ideological beliefs (e.g. in ‘free markets’) may affect implementation of the option | **[Building on traditional practices] Sood Indonesia:** The campaing build on the traditional cultural practice which embodies shared responsibility central to Indonesian value of community help |
|  | Short-term thinking | Implementation of the option may be opposed if its benefits are likely to occur beyond the time horizon of decision makers (e.g. after the next election) | **[Short term project with long term aims] Skinner**: It was, in effect, a short-term project, not embedded in a systematic strategy of intervention. In planning this project, for example, it was not possible to locate details of any similar projects which may have been undertaken, nor was the project initially seen as ongoing. No record, published or unpublished, could be found of similar activity. The time-limited nature of the project caused considerable distress to the planners and implementers when they realised that the work they had done was going to cease as the project ended. They were particularly concerned at the loss of the midwifery workforce, either to unemployment or back into the private sector. It proved challenging for the Kampong Chhnang staff to get ongoing funding for further production of the posters or to continue with community engagement. What did eventuate however was a series of requests from other districts, some of which were managed by NGOs, for use of the posters and the training packages. |
|  | Contracts | Contracts with service providers or enforcement of contracts may not be adequate to ensure implementation of the option or the types of effective care at which it is targeted | NA |
|  | Legislation or regulations | Changes to legislation or regulations, including those that are general (e.g. regulating government contracts, regulating working conditions) and those that are specific to the health system (e.g. licensing health professionals) may be needed | **[Government decisions affecting intervention] Skinner**: The fact that many of the midwives in this study had stopped being paid from Government funds part way through the year as they had run out of money reinforced the need to seek private work, undermining the effectiveness of health centre care.  **[Cost-sharing policy ended] FCI KEN** cost-sharing policy had officially ended. In addition, the overall costs of care increased; the mean costs of normal delivery care represents approximately 17% of mean monthly household income while the mean costs of complicated delivery care increased by 32%, from 1,791 Ksh, to 2,363 Ksh—an amount that could potentially result in household impoverishment. These high costs of care-seeking may be an important barrier to the use of skilled care during childbirth. |
|  | Donor policies | Donor policies and programmes may influence implementation | [**Lack of funding] Skinner**: The initial funding for the project only allowed for a six-month time period and it was seen as unfeasible to assess outcome in such a short time frame. The second stage of the evaluation was undertaken six months later. As the project was extended to look at outcome in particular, this was the focus of the second evaluation. As this had not been anticipated prior to the project commencing, specific pre-programme data for the participating villages had not been collected so outcome data were collated from routine health centre data, thought to be reasonably accurate |
|  | Influential people | The opinions of influential people may influence the composition of the option or implementation of it | **[Involving decision makers and influences in the family to adopt new practices] Kumar 2012/Kumar** **2008**: The approach of behavior change management sought to understand the deep-rooted community rationale for existing behaviors; to create a state of cognitive dissonance using common examples and messages (primarily through story-telling), which would encourage women to adopt improved practices; to create an alignment with scientific rationale for improved practices; to negotiate for recommended practices; and to support families in adopting new practices. This ushered a culture of inquisitiveness and “evidence-based” decision-making within families. The multilevel and inclusive strategy of integrating family members (including males), existing healthcare stakeholders, and community stakeholders within the folds of the intervention helped to create an enabling environment for changes in household behavior and shifts in social norms, and an open forum for discussion on issues related to newborn care and pregnancy. Because pregnant women in this region are usually not empowered with decision-making on maternal and newborn health, the engagement of CHWs with decisionmakers and influencers in the family may have enabled positive decision-making and action.  **[Involving important community leaders] Turan** The local community used several methods to promote ownership of this project. The local community leaders in the intervention area were consulted and involved from the initial visit for selection of the project site to the final assessment visits**.** Throughout the life of the project, efforts were made to implement the community members’ suggestions for improving the project  **[Involving celebreties] Sood Indonesia:** Each phase shared a common look featuring popular singer as the spokesperson for print material with consistent colour and scheme and logo which helped to make the campaign a brand name.  **[Involving religious leaders] Mushi:** Increased involvement of religious leaders in community health activities. A ward secretary commented “Because they are part of SMPs team, we have seen some of them conducting home visits and heard them promoting and encouraging women to deliver with skilled attendants during worship services”  **[Training MoH people as well] Awhualai/Kaharuza:** The project strengthened DHMT training and supervision capabilities. CBRHP used MOH “master trainers” in developing its training strategy. These master trainers received important training in reproductive health issues targeted by the project. They are now better trainers and ready to continue providing training in those subjects. By including MOH personnel in its supervisory activities where possible, the project has served to strengthen their abilities and skills in supervising and supporting community level activities. This is an activity which the MOH has largely neglected in the past. Their ability to continue to apply those skills to support VHWs/TBAs and community leadership structures will be an important factor in the sustainability of CBRHP activities and impact. |
|  | Corruption | Corrupt behaviour by decision makers or others may influence implementation |  |
|  | Political stability | Political instability may influence implementation | **[Political instability affecting methodlogy] Sood Nepal**: Given policital instability in Nepal in the ensuing period many SUMATA activities could only be conducted in and around district headquarters for security reasons and messages for urban audiences were developed.  [**Armed conflict] McPherson**: The coverage may have been influenced by the armed conflict between the government security forces and the insurgents.  **[Civil war] Hodgins** Nepal was in a state of civil war, which affected both intervention districts. Although during the conflict there were frequent anecdotal reports of security problems restricting access to hospitals, our study did not show any significant change in the proportion reporting inability to access health services due to security concerns. A more significant problem with regard to access to emergency care has been transport and geographic barriers, such conditions were essentially the same at baseline and endline. |
|  | Resource implications | How resource-intense an intervention is, whether it is cost–effective and whether it offers any incremental benefit are important considerations for sustainability | **[Programme costs[ Midhet**: for the IEEC intervention, the total cost of booklets, cassettes, group sessions and training of facilitators, etc., was roughly Rs. 530 (US$ 12) per woman in he target population. The average cost of all other training programs including healthcare providers, Dais (traditional birth attendants) and drivers, was Rs. 2,900 (US$ 60) per trainee. The telecommunications systems cost approximately US$ 30,000, which included four base stations with transmission towers and about 100 walkie-talkie instruments provided to drivers and Dais.  **[Costs] Turan:** The intervention was low-cost; the total cost to train the 60 Maternal Health Volunteers and maintain the intervention activities over a 2-year period was approximately $40,000.  **[Costs] Skinner:** The first six months of the project cost US$9000. There were 1780 educational interactions and 327 people participated in focus group discussions. This cost breaks down to $4.27 per interaction. Apart from preparation of the posters, the main costs were in planning and preparation and in supervision and support of a new project. If this was an established programme, it is estimated that the costs would possibly halve as overall planning and preparation costs could be minimised and super- vision requirements could be reduced. This pro- gramme would ideally be integrated alongside other primary health-care activities, thus reducing the cost further.  **[Costs] Brazier** If these additional institutional deliveries are attributed solely to the stimulus of demand for skilled care by the CMBCC activities, which cost 37.5 million CFA2, our narrow measure of incremental cost per delivery was 28 431 CFA or $164 international dollars.3 This is our favoured estimate. It compares with the average cost per delivery in Health Centres across the two districts which we estimate below to be $214 international dollars. If, however, SCI programme management costs (260.9 million CFA) are also included, the incremental cost per delivery increases markedly to 226 232 CFA or $1306 international dollars.  **[Combined package of intervention without results] Pasha** Another possibility is that although we had three intervention components, most sites appeared to give more attention to community mobilization and community birth attendant training and less to hospital staff training. Since a well-functioning hospital and a trained, motivated staff seem crucial for achieving the level of mortality reduction hoped for in this study, it may be that hospital training was insufficient. However, a substantial amount of hospital training occurred, and potential areas for improvement were made apparent to the hospital administrators and staff by the facility and mortality audits. |
|  | Sustainability | Whether implementation continues depends on whether the health benefits from the intervention are sustained; the intervention is incorporated into the health system; and capacity building in the system and the community | **[Government continuing with visual aids but use depends on commitment to pay for development and distribution] Hossain/Barbey**: The Birth Planning card used by DSI has been adopted for use by the GoB. However, sustainability of the card depends on staff remaining motivated to train clients to use it and the commitment of GoB or donors to pay for its provision to clients.  **[Continuation by staff] Skinner**: The staff in Kampong Chhnang were enthusiastic to continue the programme  **[Ongoing activities after closing] Ahuwalia**: Approximately 1 year after the CBRHP’s major interventions ceased in these communities, most of the VHWs continued to do health pro- motion by visiting pregnant women, teaching them about birth planning and danger signs, and assisting them in obtaining both prenatal and obstetric services. Local VHW associations are forming with support from local political leaders, the Ministry of Health, and CARE to sustain the work of the VHWs. The community development officers, some of who were also the master trainers, are involved in spearheading the formation of VHW organizations. Already, the VHWs, with the assistance of the local government, the Ministry of Health, and CARE have begun to organize themselves into local professional organizations. These organizations will work with their communities and with government agencies to help ensure that the VHWs have opportunities for further training and are compensated for their work. MOH is willing and capable of assuming responsibility for continued support, supervision and feedback to community agents and structures as well as its own personnel.  **[Government adoption of approach] Fonseca**: With regard to sustainability of these achievements, both the inservice training and preservice education systems have been strengthened, allowing for future generations of providers to receive high-quality training in evidence-based medicine. Fifty-seven Guatemalan clinical trainers were developed, and seven of eight nursing schools implemented revised curricula on EMNC. In addition, the Guatemalan government formally institutionalized the PQI process as the methodology that will be used to establish and maintain high-quality services at its health facilities on a national basis. |
